# Sensor Networks for Optimal Target Localization with Bearings-Only Measurements in Constrained Three-Dimensional Scenarios

**DOI:** 10.3390/s130810386

**Published:** 2013-08-12

**Authors:** David Moreno-Salinas, Antonio Pascoal, Joaquin Aranda

**Affiliations:** 1 Department of Computer Science and Automatic Control, National University Distance Education (UNED), Juan del Rosal 16, Madrid 28040, Spain; E-Mail: jaranda@dia.uned.es; 2 Institute for Systems and Robotics (ISR), Instituto Superior Tecnico (IST), Univ. Lisboa, Av. Rovisco Pais 1, Lisbon 1049-001, Portugal; E-Mail: antonio@isr.ist.utl.pt

**Keywords:** position estimation, positioning systems, estimation theory, localization, information analysis, optimization, autonomous vehicles, sensor networks

## Abstract

In this paper, we address the problem of determining the optimal geometric configuration of an acoustic sensor network that will maximize the angle-related information available for underwater target positioning. In the set-up adopted, a set of autonomous vehicles carries a network of acoustic units that measure the elevation and azimuth angles between a target and each of the receivers on board the vehicles. It is assumed that the angle measurements are corrupted by white Gaussian noise, the variance of which is distance-dependent. Using tools from estimation theory, the problem is converted into that of minimizing, by proper choice of the sensor positions, the trace of the inverse of the Fisher Information Matrix (also called the Cramer-Rao Bound matrix) to determine the sensor configuration that yields the minimum possible covariance of any unbiased target estimator. It is shown that the optimal configuration of the sensors depends explicitly on the intensity of the measurement noise, the constraints imposed on the sensor configuration, the target depth and the probabilistic distribution that defines the prior uncertainty in the target position. Simulation examples illustrate the key results derived.

## 1. Introduction

The last decade has witnessed tremendous progress in the development of marine technologies that are steadily affording scientists advanced equipment and methods for ocean exploration and exploitation. Recent advances in marine robotics, sensors, computers, communications and information systems are being applied to develop sophisticated technologies that will lead to safer, faster and far more efficient ways of exploring the ocean frontier, especially in hazardous conditions. As part of this trend, there has been a surge of interest worldwide in the development of autonomous underwater vehicles (AUVs) capable of roaming the oceans freely, collecting relevant data at an unprecedented scale. In fact, for reasons that have to do with autonomy, flexibility and the new trend in miniaturization, AUVs are steadily emerging as tools par excellence to replace remotely operated vehicles (ROVs) and also humans in the execution of many demanding tasks at sea. Furthermore, their use in collaborative tasks allows for the execution of complex missions, often with relatively simple systems; see [1] and the references therein.

Many AUV mission scenarios call for the availability of good underwater positioning systems to localize one or more vehicles simultaneously based on acoustic-related range or angle information received on board a support ship or an autonomous surface system (e.g., a number of autonomous surface vehicles equipped with acoustic receivers, moving in formation). The info thus obtained can be used to follow the state of progress of a particular mission or, if reliable acoustic modems are available, to relay it as a navigation aid to the navigation systems existent on board the AUVs. Similar comments apply to a future envisioned generation of positioning systems to aid in the tracking of one or more human divers. Inspired by related work and similar developments in ground robotics, in this paper, we address the problem of single target positioning based on measurements of the azimuth (bearing, in 2D scenarios) and elevation angles between an underwater target and a set of sensors, obtained via acoustic devices. Thus, the target position is determined with bearing-only measurements, in contrast to what is customary in marine systems, where range measurements are often used. In what follows, we will refer to these angle measurements in 3D as AE (azimuth-elevation) measurements or, for simplicity, with an obvious abuse of notation, simply as bearing measurements. Speaking in loose terms, we are interested in determining the optimal configuration (formation) of a sensor network that will, in a well-defined sense, maximize the AE-related information available for underwater target positioning. To this effect, we assume that the AE-measurements are corrupted by white Gaussian noise, the variance of which is distance-dependent. The computation of the target position may be done by resorting to triangulation algorithms, based on the nature of the measurements. See, for example, [2–4], and the references therein, for an introduction to this circle of ideas, covering both theoretical and practical aspects. We recall that the triangulation problem has been widely studied in the computer vision field and that there exist many examples of algorithms to compute the position of a target using angle measurements [5]; see [6] for an example of the design of motion-planning and sensor assignment strategies to track multiple targets with a mobile sensor network by resorting to triangulation.

Given a target localization problem, the optimal geometry of the sensor configuration depends strongly on the constraints imposed by the task itself (e.g., maximum number and type of sensors that can be used) and the environment (e.g., ambient noise). An inadequate sensor configuration may yield large localization errors. It is important to remark that even though the problem of optimal sensor placement for bearing- and/or range-based localization is of great importance, not many results are available on this topic yet. Exceptions include a series of interesting results that go back to the work of [7], where the Cramer-Rao Bound is used as an indicator of the accuracy of source position estimation, and a simple geometric interpretation of that bound is offered. In the same reference, the authors describe a solution to the problem of finding the sensor arrangements that minimize the bound, subject to geometric constraints. In [8], the problem of localization in two-dimensional (2D) scenarios is examined. The author explicitly computes the lowest possible geometric dilution of precision (GDOP) attainable from range or pseudo-range measurements to N optimally-located points and determines the corresponding regular polygon-like sensor configuration. In [9], the authors study optimal sensor placement and motion coordination strategies for mobile sensor networks. For a target tracking application with range sensors, they investigate the determinant of the Fisher Information Matrix and compute it for the 2D and 3D cases. They further characterize the global minimal of the 2D case. In [10], an iterative algorithm that places a number of sensors, so as to minimize the position error bound, is developed, yielding configurations for the optimal formation subject to several complex constraints. In [11,12], the authors characterize the relative sensor-target geometry for bearing-only localization and for Time-of-Arrival and Time-difference-of-Arrival in ℜ^2^. In these two references, the optimal Fisher Information Matrix is defined for a constant variance error model; again, the goal is to compute optimal sensor configurations. Finally, in [13], the authors deal with the problem of localizing and tracking an arbitrary number of non-cooperative targets with passive bearing measurements by using a Monte Carlo realization of a probability hypothesis density filter, but eschew the computation of optimal sensor configurations. For interesting related work, the reader is also referred to [14–19].

Motivated by previous results published in the literature, in this paper, we address the problem of finding the optimal geometric configuration of a sensor formation for the localization of an underwater target, based on AE-only measurements. The optimality conditions for a generic sensor formation are defined, and the explicit optimal geometric configuration of a sensor formation based on AE-only measurements is studied for two different scenarios:

- The case in which the sensors lie on a sphere centered at the target position, which provides a simple example of how to define optimal sensor configurations for a given set of (physical or mission-related) constraints imposed on the sensor formation.
- The application scenario in which a surface-based sensor formation is defined for the localization of an underwater target. Notice that in this scenario, the sensors are restricted to lie at the sea surface. A problem of this type was previously studied in [20], where a method to determine the optimal two dimensional spatial placement of multiple sensors participating in a robot perception task was introduced. One of the scenarios considered was that of localizing an underwater vehicle, with the locations of the acoustic receivers constrained to lie in a horizontal plane. In [21], the authors carried out an initial study of this particular application problem.

Given a localization strategy, the optimal sensor configuration can be ascertained by examining the corresponding Fisher Information Matrix (FIM) or its inverse, the so-called Cramer-Rao Bound (CRB) matrix. In this paper, we use the trace of the CRB matrix (A-optimality criterion [22]) as an indicator of the performance that is achievable with a given sensor configuration. Minimizing this quantity yields the most appropriate sensor formation geometry. It is important to remark that in many studies published in the literature on ground and marine robots, the determinant of the FIM (D-optimality criterion [22]) is often used as an indicator of the type of positioning performance that can be achieved. For the problem that we tackle in this paper, this indicator is not adequate, as will be shown in Section 6. This is a simple consequence of the fact that the AE measurements enter the FIM in such a way as to render its determinant extremely large for certain trigonometric configurations. However, the large value of the determinant is misleading, since it corresponds to close-to-singular configurations of the network [23]. This issue does not arise in 2D applications.

It is important to point out that following what is commonly reported in the literature, we start by addressing the problem of optimal sensor placement given an assumed position for the target. It may be argued that this assumption defeats the purpose of devising a method to compute the target position, for the latter is known in advance. The rationale for the problem at hand stems from the need to first fully understand the simpler situation, where the position of the target is known, and to characterize, in a rigorous manner, the types of solutions obtained for the optimal sensor placement problem. In a practical situation, the position of the target is only known with uncertainty, and this problem must be tackled directly. However, in this case, it is virtually impossible to make a general analytical characterization of the optimal solutions, and one must resort to numerical search methods. At this stage, an in-depth understanding of the types of solutions obtained for the ideal case is of the utmost importance to compute an initial guess for the optimal sensor placement algorithm adopted. These issues are rarely discussed in the literature, with the exception of [24,25]. The organization of the paper reflects this circle of ideas in that it effectively establishes the core theoretical tools to address and solve the case when there is uncertainty in the position of the underwater target.

The key contributions of the present paper are threefold: (i) global solutions to the optimal sensor configuration problem in 3D are obtained analytically in the cases where the sensor network is restricted to lie on a sphere centered at the target position or on a plane, the latter capturing the situation, where the sensors are deployed at the sea surface; (ii) in striking contrast to what is customary in the literature, where zero-mean Gaussian stochastic processes with fixed variances are assumed for the measurements, the variances are now allowed to depend explicitly on the distances between target and sensors. This allows us to explicitly address the important fact (rooted in first physics principles) that the measurement noise may increase in a nonlinear manner with distance; finally, (iii) the solutions derived are extended to the case, where *a priori* knowledge about the target in 3D is given in terms of a probability density function. In this latter scenario, an in-depth understanding of the types of solutions obtained for the ideal case of known target position is of the utmost importance to compute an initial guess for the optimal sensor placement algorithm adopted.

The document is organized as follows. Section 2 offers the reader a brief overview of the most common underwater positioning systems. Section 3 derives the FIM when the measurement noise is Gaussian, with distance-dependent variance. The optimal Fisher Information Matrix that minimizes the trace of the corresponding CRB matrix is computed in Section 4. The optimal sensor configuration is explicitly defined for the case in which the sensors lie on a sphere centered at the target position in Section 5. In Section 6, the optimal sensor placement is computed in the context of a sensor network restricted to lie on a plane, and two illustrative scenarios are shown as examples. In Section 7, the optimal sensor placement problem is solved for the case where the prior knowledge about the target in 3D is given in terms of a probability density function. Finally, the conclusions and a brief discussion of topics for further research are included in Section 8.

## 2. A Brief Overview of Underwater Positioning Systems

To better motivate and help understand the problem that is at the core of the present paper, this section affords the reader a brief overview of the most common underwater, acoustic-based positioning systems that are available commercially. The latter stand in sharp contrast to the techniques that are used on land or in the air, such as Global Positioning Systems (GPSs).

The most important features of a GPS system are its wide area coverage, the capability to provide navigation data seamlessly to multiple vehicles, the relatively low power requirements, the miniaturization of receivers and the fact that it is environmentally friendly, because its signals do not interfere significantly with the ecosystem. Typical acoustic underwater positioning systems are quite the opposite: they have reduced area coverage; they do not normally scale well for multiple vehicle operations; and they may have high power requirements, with a subsequent moderate to high impact on the environment in terms of acoustic pollution. The applications of underwater acoustic positioning systems include a wide range of scientific and commercial activities, such as biological and archaeological surveying, marine habitat mapping and gas and oil pipeline inspections, to name just a few. The systems available are quite diverse and suited for a number of different tasks. Most of them are based on the computation of the ranges or bearings (azimuth and elevation angles) between the target to be localized and a set of acoustic sensors with known positions. This is done by measuring the times of arrival (TOA) or the time differences of arrival (TDOA) of the acoustic signals that arrive at an array of sensors; see, for example, [2,12,26–28]. The most common positioning systems are reviewed next.

### Ultra Short Baseline Systems (USBL)

The main elements of a complete USBL system consist of an acoustic unit that works both as an emitter and receiver (*i.e.*, a transceiver), traditionally placed under the hull of a ship, and a transponder on the target to be positioned. In a typical mode of operation, the transceiver emits an acoustic wave, which, upon detection by the transponder, triggers the emission of an acoustic response by the latter. Because the transceiver is equipped with a phased transducer array (three or more transducers separated from each other by short distances, often with baselines less than 10 cm), it is capable of resolving both the range and bearings to the transponder. Traditionally, bearing angles are determined by computing the discrete difference in phase between the reception of the signal (emitted by the transponder) at the multiple transducers present in the transceiver. Since the position and attitude of the transceiver on the vessel are known, the position of the target can be estimated.

The accuracy with which an underwater target can be positioned is highly dependent on the installation and calibration of the transceiver, as well as on the accuracy with which the inertial position and attitude of the support ship can be determined using a GPS system and an advanced attitude and heading reference unit, respectively. In this sense, advanced signal processing techniques are required in these systems. Correct calibration of the system is crucial, for any error due to poor calibration of the USBL system will translate directly into errors on the target position estimates. USBL systems are widely used, because they are simple to operate and have relatively moderate prices. However, the resulting position estimation errors are usually greater that those found with longer baseline systems, are very sensitive to attitude errors on the transducer head and increase with the slant range between the transducer and the transponder. Thus, under certain operational conditions, USBL systems can yield large absolute target position errors.

### Short Baseline Systems (SBL)

The principle of operation of a SBL systems is identical to that of an USBL system in that it relies on the emission of acoustic signals by a unit installed on board a support ship, followed by the detection of the replies emitted by a transponder installed on the underwater target. However, unlike in the case of USBL systems, the receiving units have larger baselines that can be on the order of hundreds of meters [29]. A SBL system computes the ranges between the receivers and the underwater target and uses this information to determine the relative position between the vessel and the target. Typically, the baseline on this kind of systems is much smaller than the distance from the receivers to the target. The larger the baseline is, the better the positioning accuracy is. Thus, SBL systems are very attractive when operating from a large ship. Again, as in the case of USBL systems, absolute positioning of the target requires that the position and attitude of the vessel be known with good accuracy.

### Long Baseline System (LBLs)

Traditionally, LBL systems have been the most widely used for underwater target positioning. The key elements in an LBL system are a set of sea-floor mounted baseline transponders, with a spacing between them that can be on the order of a few kilometers. In this set-up, a target to be positioned carries a transceiver. In a typical application, the emitter interrogates the transponders sequentially. Upon detection of the incoming acoustic signal, each of the transponders replies to the target. The latter, in turn, measures the round trip travel time for each acoustic emission and, therefore, its range to each of the transponders. The position of the target can then be computed by using one of many available algorithms, which, in their essence, amount to performing some kind of trilateration [30,31]. Typically, LBL systems are used for relatively long range and wide area coverage navigation. The precision with which the target position can be computed depends on a number of factors that include the target depth, the frequency of the acoustic emissions and the size of the transponder baseline. As with USBS systems, the errors in the calibration of the transponder positions also impact directly on the accuracy of the target position estimates. The operational costs of a mission involving an LBL system are considerable, due to the cumbersome and time-consuming effort of deploying, calibrating and recovering the transponders, hence the need for improved underwater navigation solutions.

### GPS Intelligent Buoys System (GIB)

The GIB system was first introduced in [32]. The brief explanation that follows is essentially adapted from [3]. The GIB system consists of a set of surface buoys equipped with GPS receivers, submerged hydrophones and radio modems. The times of arrival of the acoustic signals emitted by a pinger installed on board an underwater target (synchronized with GPS time prior to system deployment) are recorded at the buoys and sent in real time through the radio network to a control unit, installed, for example, on board a support vessel. Here, the data containing information about the ranges between each buoy and the underwater emitter are used to compute an estimate of the target position using a trilateration algorithm. Note that, unlike in an LBL system, the position information is only available at the control unit and, therefore, the system cannot be directly applied for autonomous vehicle navigation. The GIB and alike systems are basically used to track underwater platforms. If one wishes to use them as a real-time underwater vehicle navigation aids, the need arises to use an acoustic modem to inform the underwater target about its estimated position. The advantage of this kind of systems is that the operational costs are reduced, because they eliminate the need to deploy, calibrate and recover a set of sea bottom transponders, while providing good accuracy on the order of a few meters. Typically, the surface buoys are moored or free to drift. There are also GIB systems with self-propelled buoys that can maneuver so as to establish a moving baseline. This is useful for target positioning when the latter operates at large depths.

The commercial systems for underwater target positioning described above share the fact that they rely on the propagation of acoustic signals and the computation of their times of arrival—or time differences of arrival—at a number of receivers. Using these principles, other non-standard positioning systems can, of course, be envisioned. Examples are available in the literature that show clearly that there is tremendous interest in the development of underwater positioning systems based on sensor networks. This is a very active area of research. The interested reader will find in [33] a survey on wireless sensor networks for underwater target positioning, with due account for localization algorithms suited for different practical scenarios. However, the survey in [33] does not address the problem of optimal sensor placement. In fact, the author mentions explicitly that there is a ‘lack of localization schemes for mobile networks and mobile swarms, and many challenging problems still demand prompt solutions’.

As a contribution to the above goal, the present paper offers a solution to the problem of optimal sensor placement for underwater target positioning with bearing (AE) measurements only. When compared with other possible techniques commonly used for underwater target positioning, the problem of determining the optimal sensor placement for target localization using AE-only measurements is of special interest, because no information flows from the sensor network to the target, and therefore, its solution does not require the exchange of information between the target and the sensor network. Furthermore, the clocks of the surface platforms do not need to be synchronized with those of the underwater targets. Thus, AE-based strategies allow for the sensor network to observe without being detected itself. A problem of this type was studied in [34] for an unmanned underwater vehicle tracking an underwater target while avoiding detection.

For the sake of clarity, the work is at first motivated by a specific positioning system that holds promise for practical applications and seeks inspiration from the original GIB system described before: the surface buoys are replaced by autonomous surface vehicles (ASVs), and the pinger installed on board the underwater target does not have to be synchronized with GPS time prior to system deployment. With this set-up, the system can not only compute the position of the underwater target, but also adaptively reconfigure its formation (geometric arrangement) in accordance with the estimated position of the target, target depth and the noise measurement characteristics of the acoustic sensors, so as to yield good positioning accuracy. The sensor placement solution described is, therefore, of key importance to adequately reconfigure the sensor formation in response to on line detected changes in the mission conditions. At the root of the algorithms to determine the geometric formation to be adopted as a specific mission unfolds are the optimality conditions determined analytically in the present work.

## 3. The Fisher Information Matrix and the Cramer-Rao Lower Bound

**Figure 1.**
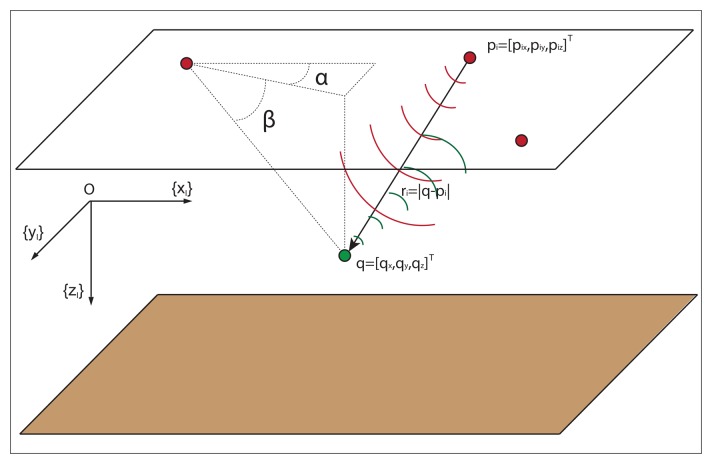
Elevation and azimuth angles measured in the inertial coordinate frame used in marine systems.

In what follows, {*I*} denotes an inertial frame with unit axis, {*x_I_*}, {*y_I_*}, and {*z_I_*} defined according to the notation that is customary in marine systems; see Figure 1. Let *q* = [*q_x_*, *q_y_*, *q_z_*]*^T^* be the location of the target to be positioned in {*I*}. Further, denote by *p_i_* = [*p_ix_*, *p_iy_*, *p_iz_*]*^T^*; *i* = 1, 2,.., *n* the position vector of the *i* − *th* acoustic sensor, also in {*I*}, where *n* is the number of sensors. Define *r̅_i_* (*q*) as the range vector from the *i* − *th* sensor to the target located at *q*, and let *r_i_*(*q*) = |*q* − *p_i_*| (abbreviated *r_i_*), where | · | denotes the Euclidean norm, denote the corresponding vector length (that is, the range between the sensor and the target).

To each of the acoustic sensors at the surface, we attach a parallel translation of {*I*}. Furthermore, for each sensor, *i* = 1, 2,.., *n*, we define *z_i_*(*q*) = (*α_i_*, *β_i_*)*^T^*, where *α_i_* and *β_i_* are the AE angles that define the direction of the target with respect to the sensor location. As it is customary, the elevation, *β*, is the angle between the range vector and the {*x_I_y_I_*} plane, while the azimuth, *α*, is the angle between the projection of the range vector in the {*x_I_y_I_*} plane and the {*x_I_*} axis; see Figure 1. Stated mathematically:

(1) $$\begin{array}{l} \alpha_{i}=\mathrm{atan}2(q_{y}-p_{iy},q_{x}-p_{ix})\\ \beta_{i}=\mathrm{atan}2(q_{z}-p_{iz},\sqrt{{\left(q_{x}-p_{ix}\right)}^{2}+{\left(q_{y}-p_{iy}\right)}^{2}}) \end{array}$$

where *atan*2 is a variation of the arctangent function to distinguish between diametrically opposite directions. We denote by *z_i_* the measurements of the actual AE angles, *α_i_* and *β_i_*, in *z_i_*(*q*), corrupted by additive noise, (*ω_αi_*, *ω_βi_*)*^T^*.

For analytical tractability, it is commonly assumed that measurement errors can be described as Gaussian, zero-mean additive noise with constant covariance. See, for example, [20], where different noise covariances are taken for different range sensors, but the covariances are constant. Clearly, the latter assumption is artificial, in view of the simple fact that the “level of noise” is distance-dependent. In this paper, in an attempt to better capture physical reality, we assume that the measurement noise can be modeled by a zero-mean Gaussian stochastic process with an added term that depends on the distance (range) between the sensor and the target. A similar error model is considered in [10] for range measurements. Stated mathematically, for an arbitrary sensor, *i*, the associated measurement noise, *ω_i_*, is given by:

(2) $$\omega_{i}={\left(\omega_{\alpha i},\omega_{\beta i}\right)}^{T}={\left(\omega_{\alpha 0}\cdot (1+\eta r_{i}^{\gamma }),\omega_{\beta 0}\cdot (1+\eta r_{i}^{\gamma })\right)}^{T}$$

where *ω_α_* and *ω_β_* are noises associated with the azimuth and elevation angle measurements, respectively, *ω_β_*_0_ and *ω_α_*_0_ are zero-mean Gaussian stochastic processes described by the probability density function, *N*(0, Σ_0_) with Σ_0_ = *σ*^2^ · *I*, *I* is the identity matrix, *r* is range and *η* and γ are modeling parameters of the distance-dependent noise component. For simplicity of exposition, and without loss of generality, the noises in the measurements of *α_i_* and *β_i_* are assumed to have identical covariances. We further assume that the covariances are identical for all sensors.

Define *z*(*q*)= (*z*_1_(*q*)*^T^*,….,*z_n_*(*q*)*^T^*)*^T^*, $z={\left(z_{1}^{T},\ldots .,z_{n}^{T}\right)}^{T}$ and $\omega ={\left(\omega_{1}^{T},\ldots .,\omega_{n}^{T}\right)}^{T}$. With this notation, the collection of all AE angle measurements obtained from all the sensors can be written as:

(3) z=z(q)+ω

or equivalently, in component form:

(4) $$z_{i}={\left(\alpha_{i},\beta_{i}\right)}^{T}+{\left(\omega_{\alpha i},\omega_{\beta i}\right)}^{T}$$

where *ω* is a Gaussian stochastic process with covariance matrix:

(5) $$\sum =\delta ((\sigma_{\alpha }^{2}\cdot {\left(1+\eta r_{1}^{\gamma }\right)}^{2},\sigma_{\beta }^{2}\cdot {\left(1+\eta r_{1}^{\gamma }\right)}^{2}),\ldots ,(\sigma_{\alpha }^{2}\cdot {\left(1+\eta r_{n}^{\gamma }\right)}^{2},\sigma_{\beta }^{2}\cdot {\left(1+\eta r_{n}^{\gamma }\right)}^{2}))$$

with Σ ∈ ℜ^2^*^nx^*^2^*^n^*, and *δ* is the operator, *diag*, that converts a vector into a square diagonal matrix, whose diagonal components are the array elements.

We assume that the reader is familiar with the concepts of Cramer-Rao Lower Bound (CRLB) and Fisher Information Matrix (FIM); see for example [35]. Stated in simple terms, the FIM captures the amount of information that measured data provide about an unknown parameter (or vector of parameters) to be estimated. Under known assumptions, the FIM is the inverse of the Cramer-Rao Bound matrix (abbreviated CRB), which lower bounds the covariance of the estimation error that can possibly be obtained with any unbiased estimator. Thus, “minimizing the CRB” may yield (by proper estimator selection) a decrease of uncertainty in the parameter estimation.

Formally, let *q̂*(*z*) be any unbiased estimator of *q*, that is, a mapping, *q̂*: ℜ*^n^*→ℜ^3^, between the observations, *z*, and the target position space, such that *E*{*q̂*} = *q* for all *q*∈ ℜ^3^, where *E*{·} denotes the average operator. Let 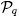
(*z*) be the likelihood function that defines the probability of obtaining the observation, *z*, given that the true target position is *q*. It is well known that under some regularity conditions on 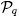
(*z*), the following inequality holds:

(6) $$\text{Cov}\{\hat{q}\}\geq \text{FIM}{\left(q\right)}^{-1}=\text{CRB}(q)$$

where:

(7) $$\text{Cov}\{\hat{q}\}=E\{(\hat{q}-q){\left(\hat{q}-q\right)}^{T}\}$$

*FIM*(*q*) (often abbreviated simply as FIM) is the Fisher Information Matrix, defined as:

(8) $$\text{FIM}(q)=E\{(\nabla_{q}\log P_{q}(z)){\left(\nabla_{q}\log P_{q}(z)\right)}^{T}\}$$

and *CRB*(*q*) is the Cramer-Rao Bound matrix. In the above, ∇*_q_* log 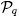
(*z*) denotes the gradient of the log of the likelihood function with respect to the unknown parameter, *q*. Taking the trace of both sides of the covariance inequality yields:

(9) $$\text{var}\{\hat{q}\}:=\text{tr}(\text{Cov}\{\hat{q}\})=\text{tr}(E\{(\hat{q}-q){\left(\hat{q}-q\right)}^{T}\})\geq \text{tr}(\text{CRB}(q))$$

which sets a lower bound on the mean-square error of any unbiased estimator.

From the above notation, following standard procedures, the FIM is computed from the likelihood function:

(10) $$P_{q}(z)=\frac{1}{{\left(2\pi \right)}^{\frac{n}{2}}{\left|\sum \right|}^{\frac{1}{2}}}\exp\left\{-\frac{1}{2}{\left(z-z(q)\right)}^{T}\sum^{-1}(z-z(q))\right\}$$

where *n* is the number of receivers, *z* is the vector of measured angles and *z*(*q*) the vector of actual angles. From Equation (8):

(11) $$\text{FIM}=E\{\nabla_{q}\log P_{q}(z)\cdot \nabla_{q}\log P_{q}{\left(z\right)}^{T}\}=F^{T}\sum^{-1}F$$

with:

(12) $$\begin{array}{cc} F= & \left[\begin{array}{ccc} \frac{-\sin(\alpha_{1})}{r_{1}\cos(\beta_{1})} & \frac{\cos(\alpha_{1})}{r_{1}\cos(\beta_{1})} & 0\\ \frac{-\sin(\beta_{1})\cos(\alpha_{1})}{r_{1}} & \frac{-\sin(\beta_{1})\sin(\alpha_{1})}{r_{1}} & \frac{-\cos(\beta_{1})}{r_{1}}\\ \vdots & \vdots & \vdots \\ \frac{-\sin(\alpha_{n})}{r_{n}\cos(\beta_{n})} & \frac{\cos(\alpha_{n})}{r_{n}\cos(\beta_{n})} & 0\\ \frac{-\sin(\beta_{n})\cos(\alpha_{n})}{r_{n}} & \frac{-\sin(\beta_{n})\sin(\alpha_{n})}{r_{n}} & \frac{-\cos(\beta_{n})}{r_{n}} \end{array}\right] \end{array}$$

where *F* ∈ ℜ^2^*^nx^*^3^, and *CRB* = *FIM*^−1^. In this context, the optimal sensor placement strategy for a single vehicle localization problem is obtained by minimizing the trace of the CRLB; this is the so-called *A-optimum design* [22]. Other indicators, like the D- or E-optimality criteria, are also very popular. The D-optimality criterion, which consists in maximizing the FIM determinant, minimizes the volume of the uncertainty ellipsoid for the target estimate, whereas the A-optimality criterion, which consists in minimizing the trace of the CRLB matrix, suppresses the average variance of the estimate, and the E-optimality design, which consists in minimizing the largest eigenvalue of the CRLB matrix, minimizes the length of the largest axis of the same ellipsoid [22].

An important advantage of D-optimality is that it is invariant under scale changes in the parameters and linear transformations of the output, whereas A-optimality and E-optimality are affected by these transformations. However, as commented upon in Section 1, the D-optimality criterion can yield to some errors, as the information in one dimension can be improved rapidly, providing a very large FIM determinant, while we can have no information in other dimensions. This problem can be avoided with the A-E-optimality criteria [23]. For this reason, in this work, the minimization of the trace of the CRLB matrix, *i.e.*, the *A-optimum design*, is used as the optimality criterion and as an indicator of the performance that is achievable with a given sensor formation.

## 4. Optimal Fisher Information Matrix

For the sake of simplicity, and without loss of generality, hereinafter, the target is considered to be placed at the origin of the inertial coordinate frame. To compute the trace of the CRB matrix, it is convenient to introduce the following three vectors in ℜ^2^*^n^*:

(13) $$\begin{array}{l} \text{X}=\left[\begin{array}{llll} \frac{F(1,1)}{\sigma (1+\eta r_{1}^{\gamma })} & \frac{F(2,1)}{\sigma (1+\eta r_{1}^{\gamma })} & \cdots & \begin{array}{ll} \frac{F(n-1,1)}{\sigma (1+\eta r_{n}^{\gamma })} & \frac{F(n,1)}{\sigma (1+\eta r_{n}^{\gamma })} \end{array} \end{array}\right]\\ ϒ=\left[\begin{array}{lllll} \frac{F(1,2)}{\sigma (1+\eta r_{1}^{\gamma })} & \frac{F(2,2)}{\sigma (1+\eta r_{1}^{\gamma })} & \cdots & \frac{F(n-1,2)}{\sigma (1+\eta r_{n}^{\gamma })} & \frac{F(n,2)}{\sigma (1+\eta r_{n}^{\gamma })} \end{array}\right]\\ \text{Z}=\left[\begin{array}{lllll} \frac{F(1,3)}{\sigma (1+\eta r_{1}^{\gamma })} & \frac{F(2,3)}{\sigma (1+\eta r_{1}^{\gamma })} & \cdots & \frac{F(n-1,3)}{\sigma (1+\eta r_{n}^{\gamma })} & \frac{F(n,3)}{\sigma (1+\eta r_{n}^{\gamma })} \end{array}\right] \end{array}$$

The latter should be viewed as vectors of a Hilbert space with elements in ℜ^2^*^n^*, endowed with a inner product structure, <, >. This allows for the computation of the length of a vector and, also, for the angle between two vectors. Namely, given X and ϒ in ℜ^2^*^n^*, then |X|^2^ = < X, X > and < X, ϒ > = |X||ϒ| cos(*θ*_Xϒ_), from which it follows that the angle, *θ*_Xϒ_, between vectors, X and ϒ, is given by *θ*_Xϒ_ = cos^−1^(< X, ϒ > /(|X||ϒ|)).

With this notation, the FIM becomes:

(14) $$\text{FIM}=\left(\begin{array}{lll} \text{X}\cdot \text{X} & \text{X}\cdot ϒ & \text{X}\cdot \text{Z}\\ \text{X}\cdot ϒ & ϒ\cdot ϒ & ϒ\cdot \text{Z}\\ \text{X}\cdot \text{Z} & ϒ\cdot \text{Z} & \text{Z}\cdot \text{Z} \end{array}\right)=\left(\begin{array}{ccc} {\left|\text{X}\right|}^{2} & \left|\text{X}\right|\left|ϒ\right|\cos(\theta_{\text{XY}}) & \left|\text{X}\right|\left|\text{Z}\right|\cos(\theta_{\text{XZ}})\\ \left|\text{X}\right|\left|ϒ\right|\cos(\theta_{\text{XY}}) & {\left|ϒ\right|}^{2} & \left|ϒ\right|\left|\text{Z}\right|\cos(\theta_{\text{YZ}})\\ \left|\text{X}\right|\left|\text{Z}\right|\cos(\theta_{\text{XZ}}) & \left|ϒ\right|\left|\text{Z}\right|\cos(\theta_{\text{YZ}}) & {\left|\text{Z}\right|}^{2} \end{array}\right)$$

from which it follows that:

(15) $$\text{tr}(\text{CRB})=\text{tr}({\text{FIM}}^{-1})=\frac{{\left|ϒ\right|}^{2}{\left|\text{Z}\right|}^{2}\left(1-\cos^{2}(\theta_{ϒ\text{Z}})\right)}{\left|\text{FIM}\right|}+\frac{{\left|\text{X}\right|}^{2}{\left|\text{Z}\right|}^{2}\left(1-\cos^{2}(\theta_{\text{XZ}})\right)}{\left|\text{FIM}\right|}+\frac{{\left|ϒ\right|}^{2}{\left|\text{X}\right|}^{2}\left(1-\cos^{2}(\theta_{\text{X}ϒ})\right)}{\left|\text{FIM}\right|}$$

where θ_Xϒ_, θ__XZ__ and θ_ϒZ_ are the angles defined by vectors X and ϒ, X and Z, and ϒ and Z, respectively, and |FIM| denotes the determinant of the FIM. Straightforward computations show that:

(16) $$|\text{FIM}|={\left|\text{X}\right|}^{2}\cdot {\left|ϒ\right|}^{2}\cdot {\left|\text{Z}\right|}^{2}\cdot \Theta$$

where:

(17) $$\Theta =1+2\cos(\theta_{ϒ\text{Z}})\cos(\theta_{\text{XZ}})\cos(\theta_{\text{X}ϒ})-\cos^{2}(\theta_{ϒ\text{Z}})-\cos^{2}(\theta_{\text{XZ}})-\cos^{2}(\theta_{\text{X}ϒ})$$

Notice how *tr*(*CRB*) has been expressed in terms of the norms of vectors X, ϒ and Z and the angles, *θ*_Xϒ_, *θ*_XZ_ and *θ*_ϒ_*_Z_*, between them. The latter depend on the variables, *α_i_, β_i_*, *r_i_*; *i* = 1, 2, …*n*, that define the positions of the sensors with respect to the target. Formally, in order to seek conditions that the optimal sensor configurations must satisfy to minimize *tr*(*CRB*), one could compute the derivatives of *tr*(*CRB*) with respect to *α_i_*, *β_i_* and *r_i_* and equate them to zero. This task is tedious and will not shed light into the form of the optimal sensor configurations. We, therefore, follow a different approach. To this effect, we rewrite Equation (15) as:

(18) $$\text{tr}(\text{CRB})=f_{\text{FIM}}^{1}+f_{\text{FIM}}^{2}+f_{\text{FIM}}^{3}=\frac{\left(1-\cos^{2}(\theta_{ϒ\text{Z}})\right)}{{\left|\text{X}\right|}^{2}\Theta }+\frac{\left(1-\cos^{2}(\theta_{\text{XZ}})\right)}{{\left|ϒ\right|}^{2}\Theta }+\frac{\left(1-\cos^{2}(\theta_{\text{X}ϒ})\right)}{{\left|\text{Z}\right|}^{2}\Theta }$$

where the definitions of $f_{\text{FIM}}^{1}$, $f_{\text{FIM}}^{2}$ and $f_{\text{FIM}}^{3}$ are obvious. We also define the auxiliary cost function:

(19) $$f^{*}(\text{CRB})=f_{\text{FIM}}^{1*}+f_{\text{FIM}}^{2*}+f_{\text{FIM}}^{3*}=\frac{1}{{\left|\text{X}\right|}^{2}}+\frac{1}{{\left|ϒ\right|}^{2}}+\frac{1}{{\left|\text{Z}\right|}^{2}}$$

Consider, now, the problem of minimizing f*(*CRB*) by proper choice of *α_i_, β_i_* and *r_i_*; *i* = 1, 2,…, *n*, and let $\alpha_{i}^{*}$, $\beta_{i}^{*}$ and $r_{i}^{*}$; *i* = 1, 2,…, *n* be a minimizing solution. Let X*, ϒ* and Z* be the corresponding vectors in ℜ^2^*^n^* . Suppose also that the corresponding angles, $\theta_{\text{X}ϒ}^{*}$, $\theta_{\text{XZ}}^{*}$ and $\theta_{ϒ\text{Z}}^{*}$, satisfy:

(20) $$\cos(\theta_{\text{X}ϒ}^{*})=\cos(\theta_{\text{XZ}}^{*})=\cos(\theta_{ϒ\text{Z}}^{*})=0$$

Then, as it will be shown next, $\alpha_{i}^{*}$, $\beta_{i}^{*}$ and $r_{i}^{*}$; *i* = 1, 2,…, *n* also minimize Equation (18). To see this, consider each of the three functions in Equation (18) independently. Take, for example, the function, $f_{\text{FIM}}^{1}$. Clearly, if the angles, $\theta_{\text{X}ϒ}^{*}$, $\theta_{\text{XZ}}^{*}$ and $\theta_{ϒ\text{Z}}^{*}$, are equal to *k*· *π*/2, where *k* is any odd natural number, then they satisfy Equation (20), and the above function takes the value, $f_{\text{FIM}}^{1}=1/{\left|\text{X}\right|}^{2}$. We now show that this is its minimum possible value. In fact, suppose that a smaller value can be obtained, which clearly requires that:

(21) $$\frac{\left(1-\cos^{2}(\theta_{ϒ\text{Z}})\right)}{\Theta }<1$$

The above inequality is equivalent to:

(22) $$0<2\cos(\theta_{ϒ\text{Z}})\cos(\theta_{\text{XZ}})\cos(\theta_{\text{X}ϒ})-\cos^{2}(\theta_{\text{XZ}})-\cos^{2}(\theta_{\text{X}ϒ})$$

Notice, however, that because: cos^2^ (*θ*_XZ_) + cos^2^ (*θ*_Xϒ_) ≥ 2 cos (*θ*_XZ_) cos (*θ*_Xϒ_) and 0 ≤ |cos (*θ*_ϒZ_)| ≤ 1, it follows that

$${\text{cos}}^{2}(\theta_{\text{XZ}})+\cos^{2}(\theta_{\text{X}ϒ})\geq 2\cos(\theta_{ϒ\text{Z}})\cos(\theta_{\text{XZ}})\cos(\theta_{\text{X}ϒ})$$

which contradicts Equation (22). Therefore:

(23) $$\frac{\left(1-\cos^{2}(\theta_{ϒ\text{Z}})\right)}{\Theta }\geq 1$$

and its minimum value of one is obtained when all the angles are equal to *k* · *π*/2, with *k* being an odd natural number. By applying the same reasoning to the other terms in the trace of the CRB in Equation (18), it follows, under the assumptions stated, that the optimal FIM is a diagonal matrix, that is:

(24) $$\text{FIM}=(\begin{array}{ccc} \text{XX} & \text{X}ϒ & \text{XZ}\\ \text{X}ϒ & ϒϒ & ϒ\text{Z}\\ \text{XZ} & ϒ\text{Z} & \text{ZZ} \end{array})=\overset{n}{\underset{i=1}{\text{∑}}}[\begin{array}{ccc} A_{i} & 0 & 0\\ 0 & B_{i} & 0\\ 0 & 0 & C_{i} \end{array}]$$

with:

$$\begin{array}{l} A_{i}=\frac{\sin^{2}(\alpha_{i})}{r_{i}^{2}\cos^{2}(\beta_{i})\cdot \sigma^{2}\cdot {\left(1+\eta r_{i}^{\gamma }\right)}^{2}}+\frac{\sin^{2}(\beta_{i})\cos^{2}(\alpha_{i})}{r_{i}^{2}\cdot \sigma^{2}\cdot {\left(1+\eta r_{i}^{\gamma }\right)}^{2}}\\ B_{i}=\frac{\cos^{2}(\alpha_{i})}{r_{i}^{2}\cos^{2}(\beta_{i})\cdot \sigma^{2}\cdot {\left(1+\eta r_{i}^{\gamma }\right)}^{2}}+\frac{\sin^{2}(\beta_{i})s^{2}(\alpha_{i})}{r_{i}^{2}\cdot \sigma^{2}\cdot {\left(1+\eta r_{i}^{\gamma }\right)}^{2}}\\ C_{i}=\frac{\cos^{2}(\beta_{i})}{r_{i}^{2}\cdot \sigma^{2}\cdot {\left(1+\eta r_{i}^{\gamma }\right)}^{2}} \end{array}$$

With the above assumption on the general form that the simplified FIM matrix will take, we now introduce a simple general procedure to derive conditions for optimal sensor placement that lend themselves to clear geometric interpretations. To this effect, define $A=\overset{n}{\underset{i=1}{\text{∑}}}A_{i}$, $B=\overset{n}{\underset{i=1}{\text{∑}}}B_{i}$, $C=\overset{n}{\underset{i=1}{\text{∑}}}C_{i}$. With this notation, the problem at hand can be converted into that of computing:

(25) $${\bar{p}}^{*}=\arg\min_{\bar{p}}\text{tr}(\text{CRB})=\arg\min_{\bar{p}}\text{tr}({\text{FIM}}^{-1})=\arg\min_{\bar{p}}(\frac{1}{A}+\frac{1}{B}+\frac{1}{C})$$

where $\bar{p}={\left[p_{1}^{T},\ldots ,p_{n}^{T}\right]}^{T}$ and p̅* are the optimal sensor positions expressed in spherical coordinates, that is, $P_{i}^{T}=[\alpha_{i},\beta_{i},r_{i}]$. Notice that the sensor positions, p̅* , must satisfy the additional constraint imposed by inequality (20), *i.e.*, the angles, *θ*_XY_*, θ*xz and *θ*_YZ_ must be equal to *k* · *π*/2, for some odd natural number, *k*, which, as explained, makes the off-diagonal elements of Equation (24) equal to zero.

Formally, the conditions that an optimal sensor configuration must satisfy may now be obtained by computing the derivatives of Equation (25) with respect to *α_i_*, *β_i_* and *r_i_*; *i* = 1, 2,…, *n* and equating them to zero. The candidate solutions must also satisfy Equation (20). This will naturally yield multiple optimal sensor configurations for single target positioning if no extra constraints are placed on the sensor configuration. To make the problem tractable, it is, therefore, important to impose configuration constraints rooted in operational considerations. In what follows, the methodology adopted is illustrated with two representative design examples: (i) first, by considering that the sensors are restricted to lie at the same distance from the target, that is, *r_i_* = *r* for all *i* = 1,⋯, *n;* and (ii) second, by considering that the sensors are restricted to lie in a horizontal plane, *i.e.*, *q_z_* − *p_iz_* = *q_z_*, where *q_z_* is the target depth and *p_iz_* = 0. The latter example captures the very important situation where the sensors are placed at the sea surface. The procedure adopted can, of course, be used to deal with other types of constraints on sensor placement.

## 5. Sensors Placed at a Fixed Distance from the Target

This section shows how the incorporation of physical or mission-related constraints on the positions of the sensors leads to a methodology to determine a solution to the optimal sensor placement that eschews tedious computations and lends itself to a simple geometric interpretation. To this effect, we consider the situation where all the sensors are placed on a sphere centered at the target position, that is, the distances from the sensors to the target are equal. With this assumption, *r_i_* = *r*; *i* = 1,⋯, *n*, where *r* is the radius of the sphere. In this case, the diagonal elements of the optimal Fisher Information Matrix Equation (24) can be written as:

(26) $$\begin{array}{l} A=\frac{1}{r^{2}\cdot \sigma^{2}\cdot {\left(1+\eta r^{\gamma }\right)}^{2}}\overset{n}{\underset{i=1}{\text{∑}}}\left(\frac{\sin^{2}(\alpha_{i})}{\cos^{2}(\beta_{i})}+\sin^{2}(\beta_{i})\cos^{2}(\alpha_{i})\right)=\Gamma A*\\ B=\frac{1}{r^{2}\cdot \sigma^{2}\cdot {\left(1+\eta r^{\gamma }\right)}^{2}}\overset{n}{\underset{i=1}{\text{∑}}}\left(\frac{\cos^{2}(\alpha_{i})}{\cos^{2}(\beta_{i})}+\sin^{2}(\beta_{i})\sin^{2}(\alpha_{i})\right)=\Gamma B*\\ C=\frac{1}{r^{2}\cdot \sigma^{2}\cdot {\left(1+\eta r^{\gamma }\right)}^{2}}\overset{n}{\underset{i=1}{\text{∑}}}\cos^{2}(\beta_{i})=\Gamma C* \end{array}$$

where $\Gamma =\frac{1}{r^{2}\cdot \sigma^{2}\cdot {\left(1+\eta r^{\gamma }\right)}^{2}}$ is constant and the same for all sensors in the formation, and A*, B*, C* are defined in the obvious manner. With the notation introduced, the problem of optimal sensor placement can be cast in the form of finding a vector, p̅* such that:

(27) $${\bar{p}}^{*}=\arg\underset{\bar{p}}{\min}\text{tr}(\text{CRB})=\arg\underset{\bar{p}}{\min}\left(\frac{1}{A^{*}}+\frac{1}{B^{*}}+\frac{1}{C^{*}}\right)$$

It is important to notice that for this scenario, the optimal solutions corresponding to constant or distance-dependent measurement noise covariances are identical. In fact, the solutions depend only on the azimuth and elevation angles of each sensor with respect to the target location, and the distance between the target and sensors does not affect the solutions (distance is the constraint parameter). This fact does not hold true in the practical scenario of surface sensor networks, as will be shown in Section 6, where the optimal solutions depend explicitly on the range distances between the target and sensors and on the noise model. At this point, the derivatives of Equation (27) with respect to *α_i_* and *β_i_* must be computed and equated to zero. Straightforward manipulations yield:

(28) $$\frac{\partial (\text{tr}(\text{CRB}))}{\partial \alpha_{i}}=2\cos(\alpha_{i})\sin(\alpha_{i})\cdot \left(\frac{1}{\cos^{2}(\beta_{i})}-\sin^{2}(\beta_{i})\right)(A^{*2}-B^{*2})=0$$

(29) $$\frac{\partial (\text{tr}(\text{CRB}))}{\partial \beta_{i}}=2\sin(\beta_{i})\left(\left(\frac{\sin^{2}(\alpha_{i})}{\cos^{3}(\beta_{i})}+\cos(\beta_{i})\cos^{2}(\alpha_{i})\right)\frac{1}{A^{*2}}+\frac{1}{B^{*2}}\left(\frac{\cos^{2}(\alpha_{i})}{\cos^{3}(\beta_{i})}+\cos(\beta_{i})\sin^{2}(\alpha_{i})\right)-\left(\frac{\cos(\beta_{i})}{C^{*2}}\right)\right)=2\sin(\beta_{i})\Phi =0$$

where the definition of Φ is clear from the context. By examining Equations (28) and (29), it is possible to define several configurations. For this reason, and because the purpose of this section is to derive a general methodology to obtain optimal sensor configurations under suitable constraints on sensor placement, we will illustrate the procedure by examining solutions that are relatively easy to obtain. Clearly, Equation (28) is satisfied, if at least one of the following conditions holds: (i) cos (α_i_) = 0; (ii) sin (α_i_) = 0; (iii) A*^2^ = B*^2^. Similarly, Equation (29) is satisfied if (i) Φ = 0 or (ii) sin (β_i_) = 0. The last condition is not studied in detail, because, if all the sensors are placed, such that sin (β_i_) = 0, it can be shown that the condition yields a local maximum for tr(CRB). Thus, in what follows, we consider that the optimality condition for Equation (29) is Φ = 0. However, it is important to keep in mind that alternative optimal solutions could be defined by combination of different optimal formations. Let us now examine the conditions corresponding to Equation (28).

If cos (*α_i_*) = 0 for all sensors in the formation, then this means that all sensors are placed in the same vertical plane, {*y_I_z_I_*}, and therefore, Equation (29) becomes:

(30) $$\cos^{4}(\beta_{i})=\frac{C^{*2}B^{*2}}{A^{*2}(B^{*2}-C^{*2})}$$

The above equation only holds for a single value of cos^4^ (*β_i_*), since A*^2^, B*^2^ and C*^2^ are constant for a given optimal configuration, and Equation (30) must be satisfied for every sensor in the formation. Thus, Equation (30) implies that the elevation angle for all elements of the sensor network must be ±*β*, which, together with cos (*α_i_*) = 0; *i* = 1, ⋯, *n*, defines four feasible optimal points for sensor placement. Clearly, this solution cannot be generalized for an arbitrary number of sensors. Furthermore, the analysis of *tr*(*CRB*) with the previous conditions shows that this solution yields a local maximum, and it is equivalent to having sin (*β_i_*) = 0 for *i* = 1, ⋯, *n;* thus, the solution is discarded.

Consider, now, the case where sin (*α_i_*) = 0 for each sensor in the formation. In this case, the sensors are placed in the vertical plane, {*x_I_z_I_*}, and Equation (29) yields:

(31) $$\cos^{4}(\beta_{i})=\frac{C^{*2}A^{*2}}{B^{*2}(A^{*2}-C^{*2})}$$

A reasoning similar to that used in the previous case allows for the conclusion that this solution must also be discarded.

Finally, if cos (*α_i_*) = 0 or sin (*α_i_*) = 0 holds for every sensor, the solution only defines a small number of optimal points for the sensor placement, so the solution cannot be generalized for an arbitrary number of sensors. Moreover, for this solution, A* = B*. Therefore, A* = B* is one of the conditions that an optimal sensor network must satisfy. Moreover, this solution can be easily generalized for an arbitrary number of sensors. Analyzing Equation (30) with A* = B* = D* for some D* yields:

(32) $$C^{*2}=D^{*2}\frac{\cos^{4}(\beta_{i})}{1+\cos^{4}(\beta_{i})}$$

It must be noticed that Equation (32) must hold for each and every sensor for a given optimal formation, since A*, B* and C* are constant for that given formation. Equation (32) can be rewritten as:

(33) $$C^{*2}=D^{*2}\frac{1}{1+\Omega }$$

where $\Omega =\frac{1}{\cos^{4}(\beta_{i})}$. Considering that an arbitrary sensor, *i*, can be under or above the target, the angle, *β_i_*, can take values between [*-π*/2, *π*/2]. In the interval, [−*π*/2, 0], Ω is strictly decreasing, and thus, $\frac{1}{1+\Omega }$ is strictly increasing, so that Equation (33) only holds for a single value of the elevation angle, $\beta =\beta_{1}^{*}$; the same angle for all the sensors placed under the target position. In the interval, [0,*π*/2], Ω is strictly increasing, and thus, $\frac{1}{1+\Omega }$ is strictly decreasing, so that, in the same way as before, Equation (33) only holds for a single value of the elevation angle, $\beta =\beta_{2}^{*}$; the same for all the sensors placed above the target position. Furthermore, since A*, B* and C* are fixed for a given sensor formation, then $\beta_{1}^{*}=-\beta_{2}^{*}$. It is clear that a given value of *β* defines a circumference on the sphere where the sensors lie, with the radius (and height, *q_z_ − p_iz_*) depending on the given angle *β*. Thus, from $\beta_{1}^{*}=-\beta_{2}^{*}$, the sensors are placed in two parallel planes over two circumferences centered at the target projections over these planes.

To define *β* regardless of the sensor distribution over the resulting circumferences, we proceed by adding the square root of Equation (33), with D* = A*, to the square root of Equation (33) with D* = B*. When doing so, all the terms in *α_t_* are canceled, and one obtains:

(34) $$2n\cos^{2}(\beta_{i})=(\frac{n}{\cos^{2}(\beta )}+n\sin^{2}(\beta ))\sqrt{\frac{\cos^{4}(\beta )}{1+\cos^{4}(\beta )}}$$

Equation (34) has a single valid solution, *β* = 42.40 degrees, and thus, the radius of the two parallel circumferences is equal to *r*′ = *r* · cos (*β* ), where *r* is the radius of the sphere (range distance). From the above, the values of A, B and C and, therefore, the norms of the vectors, X, ϒ and Z, are well-defined. Once these values of the norms of the vectors are well-defined, the extra conditions to be specified are that A* = B* and that the off-diagonal elements of the FIM are equal to zero (or, equivalently, cos (*θ*_Xϒ_) = cos (*θ*_XZ_) = cos (*θ*_ϒZ_) = 0); that is:

(35) $$\begin{array}{l} {\text{FIM}}_{12}=\overset{n}{\underset{i=1}{\text{∑}}}\frac{\sin(\alpha_{i})\cos(\alpha_{i})}{r_{i}^{2}\cos^{2}(\beta_{i})\cdot \sigma^{2}\cdot {\left(1+\eta r_{i}^{\gamma }\right)}^{2}}+\overset{n}{\underset{i=1}{\text{∑}}}\frac{\sin^{2}(\beta_{i})\cos(\alpha_{i})\sin(\alpha_{i})}{r_{i}^{2}\cdot \sigma^{2}\cdot {\left(1+\eta r_{i}^{\gamma }\right)}^{2}}=\left(\frac{1}{r^{2}\cos^{2}(\beta )\cdot \sigma^{2}\cdot {\left(1+\eta r^{\gamma }\right)}^{2}}+\frac{\sin^{2}(\beta )}{r^{2}\cdot \sigma^{2}\cdot {\left(1+\eta r^{\gamma }\right)}^{2}}\right)\overset{n}{\underset{i=1}{\text{∑}}}\cos(\alpha_{i})\sin(\alpha_{i})=0\\ {\text{FIM}}_{13}=\overset{n}{\underset{i=1}{\text{∑}}}\frac{\sin(\beta_{i})\cos(\beta_{i})\cos(\alpha_{i})}{r_{i}^{2}\cdot \sigma^{2}\cdot {\left(1+\eta r_{i}^{\gamma }\right)}^{2}}=\frac{\sin(\beta )\cos(\beta )}{r^{2}\cdot \sigma^{2}\cdot {\left(1+\eta r^{\gamma }\right)}^{2}}\overset{n}{\underset{i=1}{\text{∑}}}\cos(\alpha_{i})=0\\ {\text{FIM}}_{23}=\overset{n}{\underset{i=1}{\text{∑}}}\frac{\sin(\beta_{i})\cos(\beta_{i})\sin(\alpha_{i})}{r_{i}^{2}\cdot \sigma^{2}\cdot {\left(1+\eta r_{i}^{\gamma }\right)}^{2}}=\frac{\sin(\beta )\cos(\beta )}{r^{2}\cdot \sigma^{2}\cdot {\left(1+\eta r^{\gamma }\right)}^{2}}\overset{n}{\underset{i=1}{\text{∑}}}\sin(\alpha_{i})=0 \end{array}$$

A simple and elegant solution that satisfies the two above extra conditions is obtained by noticing the orthogonality relations for sines and cosines from Fourier analysis [36]:

(36) $$\begin{array}{l} \overset{n}{\underset{i=1}{\text{∑}}}\cos(\alpha_{i})=\overset{n}{\underset{i=1}{\text{∑}}}\sin(\alpha_{i})=\overset{n}{\underset{i=1}{\text{∑}}}\sin(\alpha_{i})\cos(\alpha_{i})=0\\ \overset{n}{\underset{i=1}{\text{∑}}}\cos^{2}(\alpha_{i})=\overset{n}{\underset{i=1}{\text{∑}}}\sin^{2}(\alpha_{i})=\frac{n}{2} \end{array}$$

Therefore, we can take a regularly distributed formation on the circumferences, with the sensors placed along one or both of them. Using classical terminology, the sensor formation must be first and second moment balanced. Therefore, with this configuration, the minimum trace of the CRB is obtained for this scenario.

## 6. Surface Sensor Network for Underwater Target Positioning

In real situations, the sensors cannot be placed at will, either due to physical or mission constraints. As an interesting application scenario, we tackle the case where the sensors are restricted to lie in the horizontal plane, *z* = 0, and we search for the minimum of the trace of the CRB. An explicit result will be shown that lends itself to an intuitive geometric interpretation without constraint in the number of sensors used for the network.

It is clear that the angles, *β_i_*, with *i* = 1, .., *n*, must take values between zero and *π*/2, because the sensors lie in the horizontal plane, above the target. It is also easy to check that the value of each *β_i_* determines the distance, *r_i_*, between the target and the *i* − *th* sensor, because *r_i_* = *q_z_*/*sin* (*β_i_*), where *q_z_* is the target depth. Thus, *r_i_* depends directly on *β_i_*, and therefore, the derivatives of the trace of the CRB with respect to *α_i_* and *β_i_* must be computed. Straightforward manipulations yield:

(37) $$\frac{\partial (\text{tr}(\text{CRB}))}{\partial \alpha_{i}}=2\cos(\alpha_{i})\sin(\alpha_{i})\sin^{2}(\beta_{i})\cdot \left(\frac{1}{\cos^{2}(\beta_{i})}-\sin^{2}(\beta_{i})\right)(A^{2}-B^{2})=0$$

(38) $$\begin{array}{l} \frac{\partial (\text{tr}(\text{CRB}))}{\partial \beta_{i}}=\left(\frac{\sin^{3}(\beta_{i})\sin^{2}(\alpha_{i})}{\cos^{3}(\beta_{i})}+2\sin^{3}(\beta_{i})\cos(\beta_{i})\cos^{2}(\alpha_{i})+\frac{\sin(\beta_{i})\sin^{2}(\alpha_{i})}{\cos(\beta_{i})}\right)\frac{1}{A^{2}}+\\ \frac{1}{B^{2}}\left(\frac{\sin^{3}(\beta_{i})\cos^{2}(\alpha_{i})}{\cos^{3}(\beta_{i})}+\frac{\sin(\beta_{i})\cos^{2}(\alpha_{i})}{\cos(\beta_{i})}+2\sin^{3}(\beta_{i})\cos(\beta_{i})\sin^{2}(\alpha_{i})\right)+\left(\frac{-\sin^{3}(\beta_{i})\cos(\beta_{i})+\sin(\beta_{i})\cos^{3}(\beta_{i})}{C^{2}}\right)+\\ \left[\left(\frac{\sin^{2}(\beta_{i})\sin^{2}(\alpha_{i})}{\cos^{2}(\beta_{i})}+\sin^{4}(\beta_{i})\cos^{2}(\alpha_{i})\right)\frac{1}{A^{2}}+\frac{\cos^{2}(\beta_{i})\sin^{2}(\beta_{i})}{C^{2}}+\left(\frac{\sin^{2}(\beta_{i})\cos^{2}(\alpha_{i})}{\cos^{2}(\beta_{i})}+\sin^{4}(\beta_{i})\sin^{2}(\alpha_{i})\right)\frac{1}{B^{2}}\right]\cdot \\ \frac{\eta \gamma {\left(q_{z}/\sin(\beta_{i})\right)}^{\gamma }}{\tan(\beta_{i})(1+\eta {\left(q_{z}/\sin(\beta_{i})\right)}^{\gamma })}=0 \end{array}$$

We now examine Equations (37) and (38). From Equation (37), it is easy to check that one of the following conditions must hold: (i) cos (*α_i_*) = 0; (ii) sin (*α_i_*) = 0; (iii) *A* − *B* = 0.

Following a procedure similar to that of the previous section, the analysis of Equation (38) with the previous conditions shows that if cos (*α_i_*) = 0 for each sensor in the formation, the solution is not optimal; so, this solution is discarded. The same occurs if sin (*α_i_*) = 0 for each sensor in the formation, and so, this solution is discarded, too. If cos (*α_i_*) = 0 or sin (*α_i_*) = 0 for each sensor in the formation, Equation (38) implies that the only feasible solution is that *A* = *B*. Therefore, *A* = *B* is one of the conditions that an optimal surface sensor network must satisfy. Analyzing Equation (38) with *A* = *B* = *D* yields:

(39) $$C^{2}=D^{2}\left(\frac{N_{1}+N_{2}}{M_{1}+M_{2}}\right)$$

where:

$$\begin{array}{c} N_{1}=\cos(\beta_{i})\sin^{3}(\beta_{i})-\sin(\beta_{i})\cos^{3}(\beta_{i})\\ N_{2}=-\cos^{2}(\beta_{i})\sin^{2}(\beta_{i})\frac{\eta \gamma {\left(q_{z}/\sin(\beta_{i})\right)}^{\gamma }}{\tan(\beta_{i})(1+\eta {\left(q_{z}/\sin(\beta_{i})\right)}^{\gamma })}\\ M_{1}=\frac{\sin^{3}(\beta_{i})}{\cos^{3}(\beta_{i})}+2\cos(\beta_{i})\sin^{3}(\beta_{i})+\frac{\sin(\beta_{i})}{\cos(\beta_{i})}\\ M_{2}=\frac{\eta \gamma {\left(q_{z}/\sin(\beta_{i})\right)}^{\gamma }}{\tan(\beta_{i})(1+\eta {\left(q_{z}/\sin(\beta_{i})\right)}^{\gamma })}\left(\frac{\sin^{2}(\beta_{i})}{\cos^{2}(\beta_{i})}+\sin^{4}(\beta_{i})\right) \end{array}$$

and A, B and C (and, therefore, D) are constant for a given sensor configuration. This equation allows us to determine the optimal sensor configuration for underwater target positioning when the sensors are placed in the same plane. An in-depth analysis of:

(40) $$f(\beta_{i})=\left(\frac{N_{1}+N_{2}}{M_{1}+M_{2}}\right)$$

reveals that Equation (39) can be satisfied for a maximum of two different values of *β_i_* at the same time, for given values of A, B and C. An equivalent angle, *β_i_*, for a group of sensors indicates that they are placed at points belonging to a circumference around the target projection in the plane, *z* = 0. Therefore, the sensors are placed on a circumference around the target projection if the solution is only one *β_i_* or on two concentric circumferences around the target projection if the optimal formation is defined by two different values of *β_i_*. A numerical analysis of these two possible solutions shows that the minimum trace is obtained if the sensors are all placed on the same circumference; therefore, *β_i_* = *β*. The value of *β*, and, therefore, the radius of the circumference where the sensors must be placed, can be obtained by solving Equation (39). Then, the sensors are all placed at the same distance from the target, *i.e.*, *r_i_* = *r* for *i* = 1, ⋯, *n*, and the two extra conditions defined by *A* = *B* and Equation (35) are satisfied, as in the previous example, with the orthogonality relations for sines and cosines from Fourier analysis Equation (36); so, the formation must be first and second moment balanced. Clearly, the solution depends on *β*, *q_z_* and the noise measurement model.

### 6.1. Simulation Examples with Known Target Position

Based on Equation (39), we now study two different scenarios that illustrate the potential of the methods developed for optimal sensor positioning. In the first scenario, one wishes to find the sensor configuration that yields the minimum CRB trace when the noise covariance is distance-independent, that is, *η* = 0. The second scenario shows how the optimal formation changes when the noise covariance is distance-dependent, that is, *η* ≠ 0. In the second scenario, the optimal formation depends directly on the modeling parameters, *η* and *γ*, and on the target depth, *q_z_*. The values of *q_z_* = 50 *m* and *σ* = 0.05 *rad* will be constant in the forthcoming examples. Clearly, in order for the information about the optimal configurations to be useful, one must check if the trace of the CRB matrix meets the desired specifications. To this effect, and for comparison purposes, the trace of the CRB matrix obtained for a number of hypothetical target points (based on a fixed optimal sensor configuration corresponding to a well-defined scenario) will, at times, be computed by allowing these points to be on a grid in a finite spatial region, 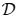. This will allow us to evaluate how adequate the sensor formation is in terms of yielding accurate localization of the real target, in comparison with the performance localization accuracy that is possible for any hypothetical target (different from the real target) positioned anywhere in 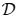. For the sake of clarity, and with an obvious abuse of notation, we will refer to that trace of the CRB, viewed as a function of its argument in 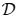, simply as *tr*(*CRB*)_
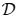
_. In this paper, 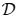 will always be a rectangle in ℜ^2^.

**Example 1:** Distance-independent covariance error.

Analyzing Equation (39) with *η* = 0 gives:

(41) $$C^{2}=D^{2}\frac{\cos^{4}(\beta )\sin^{2}(\beta )-\cos^{6}(\beta )}{1+2\cos^{4}(\beta )\sin^{2}(\beta )}$$

The value of *β*, and, therefore, the radius of the circumference where the sensors must stay, is obtained from Equation (41). The sensors are placed in a circumference centered at the target projection in the plane *z* = 0; therefore, all the range distances are the same, that is, *r_i_* = *r* for *i* = 1,…, *n*. To define *β* irrespective of the sensor distribution over the resulting circumference, we proceed by adding the square root of Equation (41), with *D* = *A*, to the square root of Equation (41) with *D* = *B*. All the terms in *α_i_* are canceled, and one obtains:

(42) $$2C=(A+B)\sqrt{\frac{\cos^{4}(\beta )\sin^{2}(\beta )-\cos^{6}(\beta )}{1+2\cos^{4}(\beta )\sin^{2}(\beta )}}$$

Straightforward computations yield:

(43) $$2\cos^{2}(\beta )\sqrt{1+2\cos^{4}(\beta )\sin^{2}(\beta )}=(1+\cos^{2}(\beta )\sin^{2}(\beta ))\sqrt{\sin^{2}(\beta )-\cos^{2}(\beta )}$$

whose only valid solution is *β* = 54.86 degrees. At this point, we may compare this optimal elevation angle with the one obtained in Section 5 for a sensor network placed over a sphere, which was equal to 42.40 degrees. We can check how the optimal elevation angle is different depending on the constraints imposed on the sensor network. This difference in the two optimal elevation angles can be negligible or very important, depending on the target depth or the limit distance considered in the mission scenario. For example, for a limit distance or depth of 50 meters, the optimal formation of Section 5 has a radius of 54.76 meters and the surface network of the example at hand, a radius of 35.19 meters. In this case the difference between formations is not important for a practical situation. However, if we consider a limit distance or target depth equal to 500 meters, the radii are 547.60 and 351.94 meters, and the difference between formations is almost 200 meters, a very significant difference. Moreover, for the scenario of Section 5, the optimal elevation angle is the same for constant and distance-dependent covariance error. In the problem at hand, the noise model is a crucial factor to determine the optimal configuration, and the solution will change depending on the noise model considered, as shown next.

**Figure 2.**
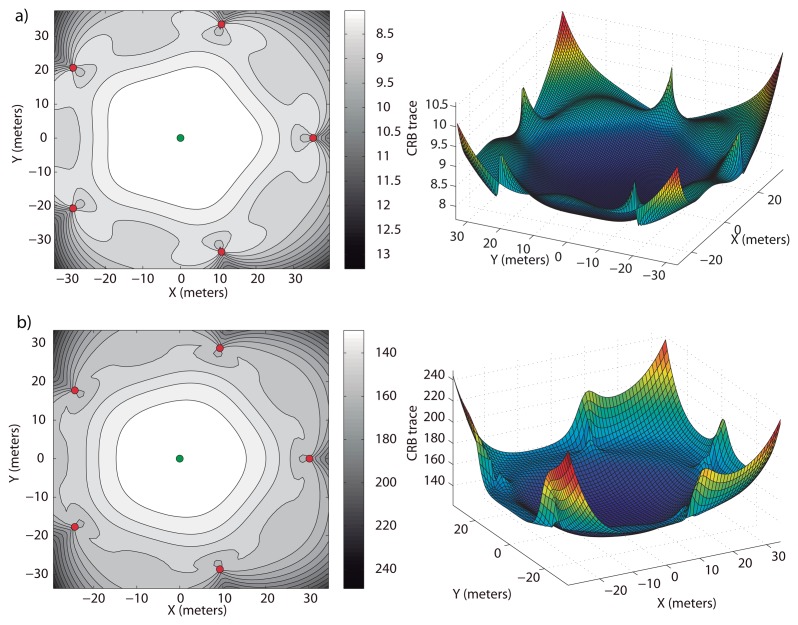
Optimal surface sensor formations for a target depth of 50 meters, *σ* = 0.05 *rad* and different values of *η*. (a) *η* = 0; (b) *η* = 0.05 and *γ* = 1. On the left-hand side, the level curves of *tr*(*CRB*)*_D_* in ℜ^2^ are shown—lighter regions indicate higher accuracy—and on the right-hand side, their magnitudes in 3D for 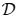.

Clearly, the optimal elevation angle, *β*, is not enough to specify the optimal location of the sensors. The extra conditions to be specified are that *A* = *B* and Equation (35). As mentioned above, these conditions are met if the sensors are first and second moment balanced; so, we can take a regularly distributed formation around the circumference. This is exactly the configuration obtained in [11] for 2D scenarios, under the explicit *a priori* condition that all sensors be placed at the same distance from the target. We thus examine the example where the sensors are regularly distributed around a circumference centered at the target projection on the surface plane. This solution can be observed in Figure 2a, where the optimal formation and the CRB trace for each point in ℜ^2^ at the target depth (*tr*(*CRB*)*_D_*) are shown on the left-hand side (lighter regions correspond to hypothetical target points with lower values of the trace of the corresponding CRB matrices). On the right-hand side of Figure 2a, it is possible to observe the value of the trace in a 3D plot and how its minimum is reached over the target position.

**Figure 3.**
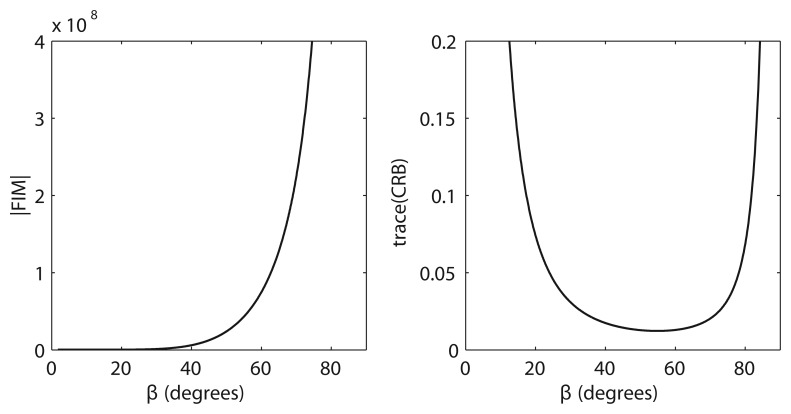
Fisher Information Matrix (FIM) determinant *versus* Cramer-Rao Bound for *β* between zero and *π*/2, considering a circular formation centered at the target projection on the surface plane.

In Figure 3, we show a comparison between the FIM determinant and the trace of the CRB for the different possible values of *β*, with *β* = *β* for all sensors, for a regular distribution of sensors around the target projection. Notice that there are configurations that yield very large values of the determinant of the FIM, but that differ from the one which provides the minimum trace of the CRB, as introduced in Section 1. Moreover, these large values correspond to configurations of the network that are clearly inadequate, e.g., they are close to configurations where all the sensors are placed at the same point, coincident with the target projection on the surface plane. It is for this reason that the trace of the CRB is used as an indicator to analyze the performance of an arbitrary formation for AE-only measurements in 3D space.

**Example 2:** Distance-dependent covariance error.

Following the reasoning of the previous example, the radius of the circumference can be obtained easily by adequately manipulating Equation (39). We can define an optimal formation where the sensors are regularly distributed around the target projection.

The only valid solution of Equation (39) yields the size of the optimal formation for single target positioning. In Figure 2b, the optimal formation is shown for a value of *η* different from zero, *η* = 0.05 and *γ* = 1. The optimal radius, that is defined by the elevation angle, *β* = 58.89 degrees, is 30.17 meters. Notice how the formation size becomes smaller when the noise between the target and sensors increases, to reduce the distance-dependent measurement noise component. The formation tends to concentrate around the projection of the target on the surface plane for increasing values of *η* and *γ* to reduce the impact of the distance-dependent measurement noise.

It is important to remark that the above values of *η* and *γ* have been chosen arbitrarily; they do not represent actual values of a possible practical scenario. The objective of the example is to show that it is critical to have an adequate noise model, for the optimal sensor formation is strongly noise-dependent. Therefore, in a practical scenario, the adequate identification of the parameters, *η* and *γ*, is of the utmost importance for the noise model to be useful.

## 7. Uncertainty in the Target Location

At this point, it is important to point out that following what is commonly reported in the literature, we have started by addressing the problem of optimal sensor placement given an assumed position for the target. In a practical situation, the position of the target is only known with uncertainty, and this problem must be tackled directly. However, in this case, it is virtually impossible to make a general analytical characterization of the optimal solutions, and one must resort to numerical search methods. At this stage, an in-depth understanding of the types of solutions obtained for the ideal case is of the utmost importance to compute an initial guess for the optimal sensor placement algorithm adopted.

The objective is to obtain a numerical solution when the target is known to lie in a well-defined uncertainty region. We assume that the uncertainty in the target position is described by a given probability distribution function, and we seek to minimize, by proper sensor placement, the average value of the trace of the CRB matrix for the target.

In what follows, *p_i_*_ξ_; *i* = 1, 2, …, *n*; *ξ* = *α*, *β*, *r* denotes the AE-measurements and range of sensor *i* located at position $p_{i}^{T}=[\alpha_{i},\beta_{i},r_{i}]$, and $\bar{p}={\left[p_{1}^{T},\ldots ,p_{n}^{T}\right]}^{T}$. We further denote by *φ*(*q*);*q* ∈ ℜ^3^a probability density function with support, *D* ∈ ℜ^3^, that describes the uncertainty in the position of the target in region *D*. With this notation, the problem of optimal sensor placement can be cast in the form of finding a vector, p̅*, such that:

(44) $${\bar{p}}^{*}=\arg\underset{\bar{p}}{\min}\int_{D}\text{tr}(\text{CRB}(\bar{p},q))\cdot \varphi (q)dq$$

where we used the notation, *CRB*(*p̅*,*q*), to clearly show the dependence of the trace of the CRB on the target and sensor locations. However, in the following, *CRB*(*p̅*, *q*) will often be denoted simply as *CRB*. In a real situation, *φ* (*q*) will depend on the type of mission carried out by the underwater target. If the target operates mostly in the center of the working area, *φ* (*q*) can, for example, assume the form of a truncated, radially-symmetric probabilistic Gaussian distribution centered at an appropriate point. On the other hand, if only the work area is known and the target can operate anywhere inside it, *φ* (*q*) can be taken as the unity function inside that area.

To proceed, *tr* (*CRB*(*p̄*, *q*)) must be computed in the equation above. At this point, it is important to remark that, given the complexity of the optimal sensor placement problem at hand, the only viable solution is a numerical one. It now remains to solve the optimization problem defined above. As explained later, we opted to use a gradient-based method to do so. To this effect, it is important to compute the derivatives of the integral in Equation (44) with respect to the sensor coordinates; that is:

(45) $$\frac{\partial }{\partial p_{i\xi }}\int_{D}\text{tr}(\text{CRB}(\bar{p},q))\varphi (q)dq$$

for *i* = 1, 2, …, *n* and *ξ* = *α*, *β*, *r*. To proceed with the computations, the integral and the derivative operations are interchanged: the derivatives are explicitly determined first, and the integration over region *D* is performed afterwards. After lengthy computations, the derivatives of *tr* (*CRB*(*p̅*, *q*)) are well-defined; see the Appendix for details.

The seemingly complex form of the derivatives, shown in the Appendix, stems from the fact that *tr*(*CRB*) is defined explicitly and from the complexity of the FIM expression, Equation (11). However, with the notation adopted, each of the derivatives of *tr*(*CRB*) with respect to the coordinates of a specific sensor can be computed in a recursive manner.

In what concerns the computation of the triple integral over the region, *D*, of interest, we opted to do it numerically using a Monte Carlo method. Finally, a solution of Equation (44) can be obtained using a gradient optimization method with the Armijo rule (see [37] and the references therein). To overcome the occurrence of local minima or the divergence of the algorithm, the initial guess in the iterative algorithm must be chosen with care. In the examples that we studied, we found it useful and expedite to adopt as an initial guess the solution for the single target positioning problem described in previous sections, with a hypothetical single target placed at the center of the work area. It is important to stress that the solution to Equation (44) depends strongly on the probability density function adopted for the target position, *q* (e.g., a truncated, radially-symmetric probabilistic Gaussian distribution or a radially-symmetric step distribution [24]).

### 7.1. Simulation Examples with Uncertain Target Location

The methodology developed is now illustrated with the help of several examples that address the problem of optimal surface sensor placement for uncertain underwater target positioning. Therefore, the main constraint imposed to the problem is that the range distances depend explicitly on the elevation angles *β_i_*, with *i* = 1, ⋯, *n*, *i.e.*, *r_i_* = *q_z_*/ sin(*β_i_*), where *q_z_* is the target depth. Different problem scenarios are studied both for constant and distance-dependent covariance error.

In this work, important practical issues related to the time required to compute the optimal sensor placement for targets lying on a region of uncertainty have not been explicitly addressed, since it is not within the scope of this work. This is a problem of considerable importance, in view of the need to compute a triple integral over a region of interest using a Monte Carlo method. For this reason, although the objective of this work is to accurately define the configurations of the optimal sensor networks, the main characteristics of the Monte Carlo computations are defined next.

For the triple integrals of each of the following examples, a set of 50,000 samples are used. The computations are carried out in a laptop Intel Core i7, with 8 Gb RAM and running an MS Windows 7 Operative System. The computation times were similar for the examples, with an average time of 128.57 s and a standard deviation of 29.31 s, for the different simulations carried out. Moreover, adding parallelism to the computations will further reduce the computing time. The above indicates that the methodology proposed for optimal sensor placement is computationally feasible.

*Scenario 1:* In this first scenario, the target is known to be working inside an area defined by a circumference with a radius of 50 meters, at a constant depth of 50 meters. A five-sensor network is used for the positioning task, and the sensors are restricted to lie in the surface plane.

**Figure 4.**
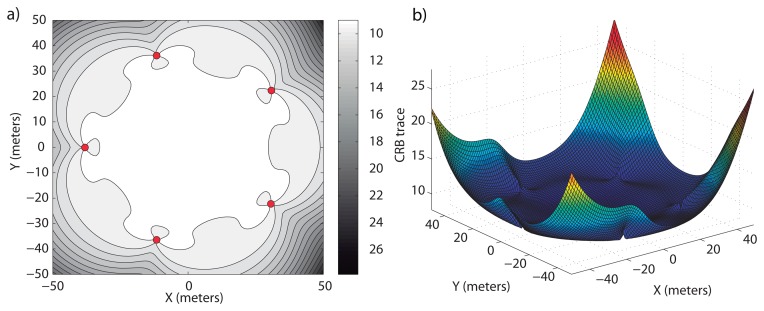
Optimal surface sensor formation for an uncertain target position at a depth of 50 meters, *σ* = 0.05 *rad* and *η* = 0. In (**a**), the level curves of *tr*(*CRB*)*_D_* in ℜ^2^ are shown and in (**b**), its magnitude in 3D for 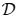.

**Example 3:** The first example of this scenario corresponds to a constant covariance positioning problem with *σ* = 0.05 *rad*. After the optimization method described above, it is found that the optimal surface formation is the one described in Figure 4. We may notice in Figure 4a how the formation keeps a regular distribution around the work area, with an optimum radius of 38.1 *m*, forming a regular pentagon, and in Figure 4b, how a homogeneous trace of the CRB matrix is obtained inside the area of interest, keeping a homogeneous accuracy. The maximum and minimum values of the CRB trace inside the area of interest are 14.01 *m*^2^ and 7.73 *m*^2^, respectively. Despite the difference between the maximum and minimum values of the CRB trace, the average value inside the work area is 8.63 *m*^2^; so, the average accuracy is close to the optimal one, and thus, for most points, the accuracy is closer to the minimum value of the CRB trace. Notice how the optimal radius becomes larger than in Example 1, where the target position was known without uncertainty.

**Figure 5.**
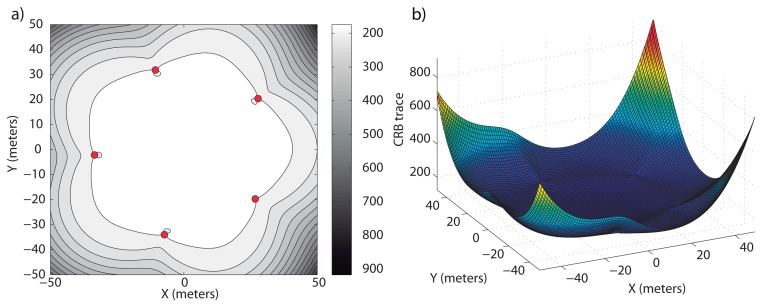
Optimal surface sensor formation for an uncertain target position at a depth of 50 meters, *σ* = 0.05 *rad* and *η* = 0.05. In (**a**) the level curves of *tr*(*CRB*)*_D_* in ℜ^2^ are shown and in (**b**), its magnitude in 3D for 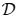.

**Example 4:** This example corresponds to a distance-dependent covariance problem, with *η* = 0.05 and *γ* = 1. We can notice in Figure 5a the difference of this optimal formation with respect to the optimal one of Example 3, shown in Figure 4a. The optimal formation is defined by a radius of 33.8 *m*, with the sensors regularly distributed around the target projection. We can notice in Figure 5b how the values of the trace of the CRB matrices are larger, due to the added distance-dependent error. The maximum and minimum values of the CRB trace inside the work area are 356.76 *m*^2^ and 124.25 *m*^2^, respectively. However, a homogeneous accuracy over the area of interest is obtained, with an average value of 161.15 *m*^2^, which shows that for most of the points of the area of interest, an accuracy close to the minimum one is obtained.

**Figure 6.**
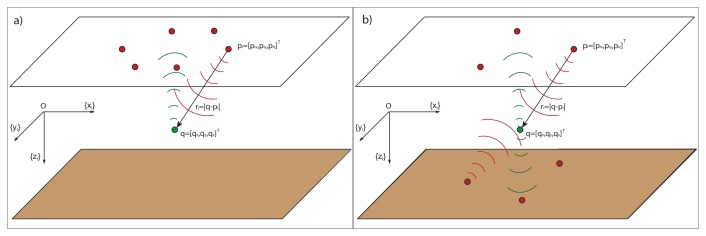
Sensor formations for an uncertainty volume of 60 × 60 × 60 *m*^3^ (**a**) surface sensor network; and (**b**) sensor network split into two formations, one at the sea surface and another at the sea bottom.

*Scenario 2:* In this second scenario, the target is placed inside an area of 60 × 60 × 60 *m*^3^ centered at the origin of the inertial coordinate frame and its center placed at 50 meters under the ocean surface and 50 meters over the ocean bottom, but there is no additional knowledge about the target position; so, the probability distribution function is a step-like distribution. The target is positioned by a six-sensor network at the sea surface, as shown in the set-up of Figure 6a. Again, both situations with constant and distance-dependent covariance are studied.

**Example 5:** This example deals with a constant covariance error with *σ* = 0.05 *rad*. No figures are shown, because it is not possible to adequately show the accuracy in a figure when a volume is studied. The optimal sensor formation that maximizes the accuracy inside the volume of interest takes a shape similar to a circumference, with an approximate radius of 41 meters. The sensor positions, in Cartesian coordinates, are shown in Table 1.

The minimum and maximum CRB trace values obtained inside the volume are *tr*(*CRB*)*_min_* = 2.44 *m*^2^ and *tr*(*CRB*)*_max_* = 18.62 *m*^2^, respectively, with an average value of *tr*(*CRB*)*_avg_* = 8.11 *m*^2^, providing a large accuracy for most points inside the region of interest.

**Example 6:** In the second example of this scenario, the error is considered to be distance-dependent, with *σ* = 0.05 *rad*, *η* = 0.1 and *γ* = 1. After the gradient optimization, the optimal sensor network is placed at the positions listed in Table 2.

We may notice how the formation is smaller than that of Example 5 to reduce the impact of the distance-dependent added error, with the network keeping a formation similar to a circumference of an approximate radius of 37 meters. The minimum and maximum CRB traces inside the volume of interest are *triCRB*)*_min_* = 49.39 *m*^2^ and *tr*(*CRB*)*_max_* = 2.17 · 10^3^*m*^2^, respectively, with an average value of *tr*(*CRB*)*_avg_* = 591.05 *m*^2^, which shows that, in this example, the accuracy is dramatically affected by the added distance-dependent error component.

*Scenario 3:* We now tackle the same situation of Scenario 2, but the sensor network can be placed in two different planes, that is, one subnetwork at the sea surface and another subnetwork at the sea bottom, shown in the set-up of Figure 6b.

**Example 7:** In this example, we consider a constant covariance measurement error, with *η* = 0 and *σ* = 0.05 *rad*. After the optimization process, in which three sensors are constrained to lie at the sea surface, *i.e.*, 50 meters above the center of the volume of interest, and the other three sensors are constrained to lie at the sea bottom, at 50 meters under the center of the volume of interest, the optimal formation is such that the sensors are placed, in Cartesian coordinates, at the positions stated in Table 3.

Notice that the formation shape, although split into two formations, is very similar to the one obtained in the previous scenario, but with an approximate radius of 26 meters. However, in this case, the minimum and maximum CRB traces are *tr*(*CRB*)*_min_* = 2.23 *m*^2^ and *tr*(*CRB*)*_max_* = 7.27 *m*^2^, respectively, with an average value of *tr*(*CRB*)*_avg_* = 5.13 *m*^2^, which shows how the accuracy, for the constant covariance case, increases when the formation consists of two formations, one at the sea surface and another at the sea bottom. Notice how the maximum value of the CRB trace is smaller with respect to Example 5 and how the average CRB trace is very close to the minimum value.

**Example 8:** Finally, this example tackles the distance-dependent covariance problem, with *σ* = 0.05 *rad*, *η* = 0.1 and *γ* = 1. In this case, the optimal formation is the one in which the sensors take the positions shown in Table 4.

Again, the formation shape is similar to that obtained in Example 6, but with an approximate radius of 22 meters. The minimum and maximum CRB trace are now *tr*(*CRB*)*_min_* = 39.36 *m*^2^ and *tr*(*CRB*)*_max_* = 414.77 *m*^2^, respectively, with an average value of *tr*(*CRB*)*_avg_* = 214.09 *m*^2^. We can notice how the maximum CRB trace is significantly reduced with respect to the value obtained in Example 6. The average value is, again, smaller, showing that a very good average accuracy is obtained inside the volume of interest. Finally, the minimum value of the CRB trace is also smaller. Thus, a more homogeneous accuracy inside the region of interest, with a significantly smaller error, is obtained when the sensors are split into two formations, one at the sea surface and the other at the sea bottom.

Therefore, for an unknown target location, it is clear that the average accuracy inside the working area is improved if we can place the sensors in two different parallel planes. This fact shows the importance of the constraints that are imposed on the sensor placement in order to define the sensor configuration that provides the largest possible accuracy in the volume of interest.

## 8. Conclusions and Future Work

We studied the problem of determining optimal configurations of sensor networks that will, in a well-defined sense, maximize the AE-related information available for underwater target positioning. To this effect, we assumed that the measurements were corrupted by white Gaussian noise, the variance of which is distance-dependent. The Fisher Information Matrix and the minimization of the trace of the CRB matrix were used to determine the optimal sensor configurations. Explicit analytical results were obtained for both distance-dependent and distance-independent noise. In the application scenario of underwater target positioning by a surface sensor network, we have shown that the optimal formation lies on a circumference around the target projection and that a “regularly distributed formation” around this target provides an optimal configuration, the size of which depends on the measurement noise model and the target depth. The methodology was then extended to deal with uncertainty in the target location, because in a practical situation, the target position is only known with uncertainty. Simulation examples illustrated the concepts developed in different application scenarios, showing that the optimal configuration of the sensors depends explicitly on the intensity of the measurement noise, the constraints imposed on the sensor configuration, the target depth and the probabilistic distribution that defines the prior uncertainty in the target position.

Future work will aim at: (i) extending the methodology developed to deal with more than one target simultaneously; and (ii) studying the performance of the algorithms for optimal sensor configuration placement developed herein, together with selected algorithms for target tracking and cooperative sensor motion control.

## Figures and Tables

**Table 1.** Optimal sensor positions for constant covariance.

	***p*_1_**	***p*_2_**	***p*_3_**	***p*_4_**	***p*_5_**	***p*_6_**
{*x_I_*}(*m*)	35.48	0.07	−35.33	−35.3	0.07	35.48
{*y_I_*}(*m*)	20.37	40.80	20.37	−20.52	−40.96	−20.52
{*z_I_*}(*m*)	50	50	50	50	50	50

**Table 2.** Optimal sensor positions for *σ* = 0.05 *rad*, *η* = 0.1 and *γ* = 1.

	***p*_1_**	***p*_2_**	***p*_3_**	***p*_4_**	***p*_5_**	***p*_6_**
{*x_I_*}(*m*)	32.76	0.04	−32.69	−32.68	0.04	32.76
{*y_I_*}(*m*)	18.91	37.80	18.91	−18.87	−37.77	−18.87
{*z_I_*}(*m*)	50	50	50	50	50	50

**Table 3.** Optimal sensor positions for constant covariance.

	***p*_1_**	***p*_2_**	***p*_3_**	***p*_4_**	***p*_5_**	***p*_6_**
{*x_I_*}(*m*)	21.98	−0.04	−22.15	−22.23	−0.08	22.14
{*y_I_*}(*m*)	12.841	25.68	12.84	−12.7	−25.38	−12.74
{*z_I_*}(*m*)	−50	50	−50	50	−50	50

**Table 4.** Optimal sensor positions for *σ* = 0.05 *rad*, *η* = 0.1 and *γ* = 1.

	***p*_1_**	***p*_2_**	***p*_3_**	***p*_4_**	***p*_5_**	***p*_6_**
{*x_I_*}(*m*)	19.74	0.14	−19.28	−19.41	0.21	19.70
{*y_I_*}(*m*)	11.16	22.66	11.14	−11.16	−22.66	−11.14
{*z_I_*}(*m*)	−50	50	−50	50	−50	50

## Appendix

This Appendix contains the derivatives of the trace of the CRB with respect to the angles, *α_i_* and *β_i_*, of the *i – th* acoustic sensor. These derivatives are used in Section 7 for the gradient optimization algorithm to determine the optimal sensor placement for single target positioning with AE-measurements with uncertainty in the target location. For the sake of completeness, the FIM for AE-measurements is defined again:

(46) $$\text{FIM}=E\{\nabla_{q}\log p_{q}\cdot \nabla_{q}\log p_{q}^{T}\}=F^{T}\sum^{-1}F$$

with:

(47) $$\sum =\delta \left({\left(\sigma_{\alpha }^{2}\cdot {\left(1+\eta r^{\gamma }\right)}^{2},\sigma_{\beta }^{2}\cdot (1+\eta r^{\gamma })\right)}^{T}\right)$$

and:

(48) $$\begin{array}{cc} F= & \left[\begin{array}{ccc} \frac{-\sin(\alpha_{1})}{r_{1}\cos(\beta_{1})} & \frac{\cos(\alpha_{1})}{r_{1}\cos(\beta_{1})} & 0\\ \frac{-\sin(\beta_{1})\cos(\alpha_{1})}{r_{1}} & \frac{-\sin(\beta_{1})\sin(\alpha_{1})}{r_{1}} & \frac{-\cos(\beta_{1})}{r_{1}}\\ \vdots & \vdots & \vdots \\ \frac{-\sin(\alpha_{n})}{r_{n}\cos(\beta_{n})} & \frac{\cos(\alpha_{n})}{r_{n}\cos(\beta_{n})} & 0\\ \frac{-\sin(\beta_{n})\cos(\alpha_{n})}{r_{n}} & \frac{-\sin(\beta_{n})\sin(\alpha_{n})}{r_{n}} & \frac{-\cos(\beta_{n})}{r_{n}} \end{array}\right] \end{array}$$

where *F* ∈ ℜ^2^*^nx^*^3^, Σ ∈ ℜ^2^*^nx^*^2^*^n^* and *CRB* = *FIM^−^*^1^. The sensors are considered to be placed at the sea surface, so that the range distance, *r_i_*, of the *i* − *th* sensor can be rewritten as *r_i_* = *q_z_*/ sin(*β_i_*), where *q_z_* is the target depth and *β_i_* is the elevation angle, as described in Section 3. For the sake of simplicity, the FIM described in Equation (46) is rewritten as:

(49) $$\text{FIM}=[\begin{array}{ccc} {\text{FIM}}_{11} & {\text{FIM}}_{12} & {\text{FIM}}_{13}\\ {\text{FIM}}_{12} & {\text{FIM}}_{22} & {\text{FIM}}_{23}\\ {\text{FIM}}_{13} & {\text{FIM}}_{23} & {\text{FIM}}_{33} \end{array}]$$

where:

$$\begin{array}{c} {\text{FIM}}_{11}=\overset{n}{\underset{i=1}{\text{∑}}}\left(\frac{\sin^{2}(\alpha_{i})\sin^{2}(\beta_{i})}{q_{z}^{2}\cos^{2}(\beta_{i})\cdot \sigma^{2}\cdot {\left(1+\eta {\left(\frac{q_{z}}{\sin(\beta_{i})}\right)}^{\gamma }\right)}^{2}}+\frac{\sin^{4}(\beta_{i})\cos^{2}(\alpha_{i})}{q_{z}^{2}\cdot \sigma^{2}\cdot {\left(1+\eta {\left(\frac{q_{z}}{\sin(\beta_{i})}\right)}^{\gamma }\right)}^{2}}\right)\\ {\text{FIM}}_{22}=\overset{n}{\underset{i=1}{\text{∑}}}\left(\frac{\cos^{2}(\alpha_{i})\sin^{2}(\beta_{i})}{q_{z}^{2}\cos^{2}(\beta_{i})\cdot \sigma^{2}\cdot {\left(1+\eta {\left(\frac{q_{z}}{\sin(\beta_{i})}\right)}^{\gamma }\right)}^{2}}+\frac{\sin^{4}(\beta_{i})\sin^{2}(\alpha_{i})}{q_{z}^{2}\cdot \sigma^{2}\cdot {\left(1+\eta {\left(\frac{q_{z}}{\sin(\beta_{i})}\right)}^{\gamma }\right)}^{2}}\right)\\ {\text{FIM}}_{33}=\overset{n}{\underset{i=1}{\text{∑}}}\left(\frac{\cos^{2}(\beta_{i})\sin^{2}(\beta_{i})}{q_{z}^{2}\cdot \sigma^{2}\cdot {\left(1+\eta {\left(\frac{q_{z}}{\sin(\beta_{i})}\right)}^{\gamma }\right)}^{2}}\right)\\ {\text{FIM}}_{12}=\overset{n}{\underset{i=1}{\text{∑}}}\left(\frac{\sin(\alpha_{i})\cos(\alpha_{i})\sin^{2}(\beta_{i})}{q_{z}^{2}\cos^{2}(\beta_{i})\cdot \sigma^{2}\cdot {\left(1+\eta {\left(\frac{q_{z}}{\sin(\beta_{i})}\right)}^{\gamma }\right)}^{2}}+\frac{\sin^{4}(\beta_{i})\cos(\alpha_{i})\sin(\alpha_{i})}{q_{z}^{2}\cdot \sigma^{2}\cdot {\left(1+\eta {\left(\frac{q_{z}}{\sin(\beta_{i})}\right)}^{\gamma }\right)}^{2}}\right)\\ {\text{FIM}}_{13}=\overset{n}{\underset{i=1}{\text{∑}}}\left(\frac{\cos(\beta_{i})\sin^{3}(\beta_{i})\cos(\alpha_{i})}{q_{z}^{2}\cdot \sigma^{2}\cdot {\left(1+\eta {\left(\frac{q_{z}}{\sin(\beta_{i})}\right)}^{\gamma }\right)}^{2}}\right)\\ {\text{FIM}}_{23}=\overset{n}{\underset{i=1}{\text{∑}}}\left(\frac{\cos(\beta_{i})\sin^{3}(\beta_{i})\sin(\alpha_{i})}{q_{z}^{2}\cdot \sigma^{2}\cdot {\left(1+\eta {\left(\frac{q_{z}}{\sin(\beta_{i})}\right)}^{\gamma }\right)}^{2}}\right) \end{array}$$

With the above notation, the trace of the CRB matrix yields:

(50) $$\begin{array}{ll} \text{tr}(\text{CRB}) & =\frac{{\text{FIM}}_{22}{\text{FIM}}_{33}-FIM_{23}^{2}}{\left|\text{FIM}\right|}\\ & +\frac{{\text{FIM}}_{11}{\text{FIM}}_{33}-{\text{FIM}}_{13}^{2}}{\left|\text{FIM}\right|}+\frac{{\text{FIM}}_{11}{\text{FIM}}_{22}-FIM_{12}^{2}}{\left|\text{FIM}\right|} \end{array}$$

and its derivatives with respect to *ξ*, where *ξ* = *α_i_*, *β_i_*, become:

(51) $$\begin{array}{ll} \frac{\partial \text{tr}(\text{CRB})}{\partial \xi_{i}} & =(\frac{\partial {\text{FIM}}_{22}}{\partial \xi_{i}}{\text{FIM}}_{33}+\frac{\partial {\text{FIM}}_{33}}{\partial \xi_{i}}{\text{FIM}}_{22}-2\frac{\partial {\text{FIM}}_{23}}{\partial \xi_{i}}{\text{FIM}}_{23}\\ & +\frac{\partial {\text{FIM}}_{11}}{\partial \xi_{i}}{\text{FIM}}_{33}+\frac{\partial {\text{FIM}}_{33}}{\partial \alpha_{i}}{\text{FIM}}_{11}-2\frac{\partial {\text{FIM}}_{13}}{\partial \alpha_{i}}{\text{FIM}}_{13}+\frac{\partial {\text{FIM}}_{11}}{\partial \xi_{i}}{\text{FIM}}_{22}\\ & +\frac{\partial {\text{FIM}}_{22}}{\partial \xi_{i}}{\text{FIM}}_{11}-2\frac{\partial {\text{FIM}}_{12}}{\partial \xi_{i}}{\text{FIM}}_{12}){\left|\text{FIM}\right|}^{-1}+\text{tr}(\text{CRB})\cdot \frac{\partial \left|\text{FIM}\right|}{\partial \xi_{i}}{\left|\text{FIM}\right|}^{-1} \end{array}$$

where:

(52) $$\begin{array}{ll} \frac{\partial \left|\text{FIM}\right|}{\partial \xi_{i}}= & \frac{\partial {\text{FIM}}_{11}}{\partial \xi_{i}}{\text{FIM}}_{22}{\text{FIM}}_{33}+\frac{\partial {\text{FIM}}_{22}}{\partial \xi_{i}}{\text{FIM}}_{11}{\text{FIM}}_{33}+\frac{\partial {\text{FIM}}_{33}}{\partial \xi_{i}}{\text{FIM}}_{22}{\text{FIM}}_{11}\\ & +2\frac{\partial {\text{FIM}}_{12}}{\partial \xi_{i}}{\text{FIM}}_{23}{\text{FIM}}_{13}+2\frac{\partial {\text{FIM}}_{13}}{\partial \xi_{i}}{\text{FIM}}_{23}{\text{FIM}}_{12}+2\frac{\partial {\text{FIM}}_{23}}{\partial \xi_{i}}{\text{FIM}}_{12}{\text{FIM}}_{13}\\ & -\frac{\partial {\text{FIM}}_{11}}{\partial \xi_{i}}FIM_{23}^{2}-2\frac{\partial {\text{FIM}}_{23}}{\partial \xi_{i}}{\text{FIM}}_{23}{\text{FIM}}_{11}-\frac{\partial {\text{FIM}}_{22}}{\partial \xi_{i}}{\text{FIM}}_{13}\\ & -2\frac{\partial {\text{FIM}}_{13}}{\partial \xi_{i}}{\text{FIM}}_{13}{\text{FIM}}_{22}-\frac{\partial {\text{FIM}}_{33}}{\partial \xi_{i}}{\text{FIM}}_{12}-2\frac{\partial {\text{FIM}}_{12}}{\partial \xi_{i}}{\text{FIM}}_{12}{\text{FIM}}_{33} \end{array}$$

To finalize with the analysis of the derivatives of the trace of the CRB matrix with respect to the angles, *α_i_* and *β_i_*, it only remains to define the derivatives of the elements of the FIM with respect to these angles, so that the whole derivatives are defined explicitly. We define, next, the derivative of each FIM component with respect to *α_i_* and *β_i_*, respectively.

$$\begin{array}{c} \frac{\partial {\text{FIM}}_{11}}{\partial \alpha_{i}}=\left(\frac{2\sin(\alpha_{i})\cos(\alpha_{i})\sin^{2}(\beta_{i})}{\cos^{2}(\beta_{i})}-2\sin(\alpha_{i})\cos(\alpha_{i})\sin^{4}(\beta_{i})\right)\cdot \Gamma_{i}^{2}\\ \frac{\partial {\text{FIM}}_{11}}{\partial \beta_{i}}=\left(\frac{2\sin(\beta_{i})\sin^{2}(\alpha_{i})}{\cos(\beta_{i})}-\frac{2\sin^{3}(\beta_{i})\sin^{2}(\alpha_{i})}{\cos^{3}(\beta_{i})}+4\sin^{3}(\beta_{i})\cos(\beta_{i})\cos^{2}(\alpha_{i})\right)\cdot \Gamma_{i}^{2}\\ +\left(\frac{\sin^{2}(\alpha_{i})\sin^{2}(\beta_{i})}{\cos^{2}(\beta_{i})}+\sin^{4}(\beta_{i})\cos^{2}(\alpha_{i})\right)\cdot \frac{\partial \Gamma_{i}^{2}}{\partial \beta_{i}}\\ \frac{\partial {\text{FIM}}_{22}}{\partial \alpha_{i}}=\left(-\frac{2\sin(\alpha_{i})\cos(\alpha_{i})\sin^{2}(\beta_{i})}{\cos^{2}(\beta_{i})}+2\sin(\alpha_{i})\cos(\alpha_{i})\sin^{4}(\beta_{i})\right)\cdot \Gamma_{i}^{2}\\ \frac{\partial {\text{FIM}}_{22}}{\partial \beta_{i}}=\left(\frac{2\sin(\beta_{i})\cos^{2}(\alpha_{i})}{\cos(\beta_{i})}-\frac{2\sin^{3}(\beta_{i})\cos^{2}(\alpha_{i})}{\cos^{3}(\beta_{i})}+4\sin^{3}(\beta_{i})\cos(\beta_{i})\cos^{2}(\alpha_{i})\right)\cdot \Gamma_{i}^{2}\\ +\left(\frac{\cos^{2}(\alpha_{i})\sin^{2}(\beta_{i})}{\cos^{2}(\beta_{i})}+\sin^{4}(\beta_{i})\cos^{2}(\alpha_{i})\right)\cdot \frac{\partial \Gamma_{i}^{2}}{\partial \beta_{i}}\\ \frac{\partial {\text{FIM}}_{33}}{\partial \alpha_{i}}=0\\ \frac{\partial {\text{FIM}}_{33}}{\partial \beta_{i}}=\left(2\sin(\beta_{i})\cos^{3}(\beta_{i})-2\cos(\beta_{i})\sin^{3}(\beta_{i})\right)\cdot \Gamma_{i}^{2}+(\cos^{2}(\beta_{i})\sin^{2}(\beta_{i}))\cdot \frac{\partial \Gamma_{i}^{2}}{\partial \beta_{i}}\\ \frac{\partial {\text{FIM}}_{12}}{\partial \alpha_{i}}=\left(\frac{\left(\cos^{2}(\alpha_{i})-\sin^{2}(\alpha_{i}))\sin^{2}(\beta_{i}\right)}{\cos^{2}(\beta_{i})}+(\cos^{2}(\alpha_{i})-\sin^{2}(\alpha_{i}))\sin^{4}(\beta_{i})\right)\cdot \Gamma_{i}^{2}\\ \frac{\partial {\text{FIM}}_{12}}{\partial \beta_{i}}=\Gamma_{i}^{2}\cdot (\frac{2\sin(\beta_{i})\sin(\alpha_{i})\cos(\alpha_{i})}{\cos(\beta_{i})}+4\sin^{3}(\beta_{i})\cos(\beta_{i})\cos(\alpha_{i})\sin(\alpha_{i})\\ -\frac{2\sin^{3}(\beta_{i})\sin(\alpha_{i})\cos(\alpha_{i})}{\cos^{3}(\beta_{i})})+\sin(\alpha_{i})\cos(\alpha_{i})\left(\frac{\sin^{2}(\beta_{i})}{\cos^{2}(\beta_{i})}+\sin^{4}(\beta_{i})\right)\frac{\partial \Gamma_{i}^{2}}{\partial \beta_{i}}\\ \frac{\partial {\text{FIM}}_{13}}{\partial \alpha_{i}}=-\sin^{3}(\beta_{i})\cos(\beta_{i})\sin(\alpha_{i})\cdot \Gamma_{i}^{2}\\ \frac{\partial {\text{FIM}}_{13}}{\partial \beta_{i}}=\cos(\alpha_{i})(3\sin^{2}(\beta_{i})\cos^{2}(\beta_{i})-\sin^{4}(\beta_{i}))\cdot \Gamma_{i}^{2}+\sin^{3}(\beta_{i})\cos(\beta_{i})\cos(\alpha_{i})\cdot \frac{\partial \Gamma_{i}^{2}}{\partial \beta_{i}}\\ \frac{\partial {\text{FIM}}_{23}}{\partial \alpha_{i}}=-\sin^{3}(\beta_{i})\cos(\beta_{i})\sin(\alpha_{i})\cdot \Gamma_{i}^{2}\\ \frac{\partial {\text{FIM}}_{23}}{\partial \beta_{i}}=\sin(\alpha_{i})(3\sin^{2}(\beta_{i})\cos^{2}(\beta_{i})-\sin^{4}(\beta_{i}))\cdot \Gamma_{i}^{2}+\sin^{3}(\beta_{i})\cos(\beta_{i})\sin(\alpha_{i})\cdot \frac{\partial \Gamma_{i}^{2}}{\partial \beta_{i}} \end{array}$$

and finally:

$$\frac{\partial \Gamma_{i}^{2}}{\partial \beta_{i}}=\frac{2\eta \gamma {\left(\frac{q_{z}}{\sin(\beta_{i})}\right)}^{\gamma }}{q_{z}^{2}\sigma^{2}\tan(\beta_{i}){\left(1+\eta {\left(\frac{q_{z}}{\sin(\beta_{i})}\right)}^{\gamma }\right)}^{3}}$$

Therefore, the derivatives of the CRB trace with respect to *α_i_* and *β_i_* are well-defined and can be used explicitly for the gradient optimization algorithm to define optimal sensor networks for underwater positioning with uncertain target location.
